# Extreme ^13^C depletion of carbonates formed during oxidation of biogenic methane in fractured granite

**DOI:** 10.1038/ncomms8020

**Published:** 2015-05-07

**Authors:** Henrik Drake, Mats E. Åström, Christine Heim, Curt Broman, Jan Åström, Martin Whitehouse, Magnus Ivarsson, Sandra Siljeström, Peter Sjövall

**Affiliations:** 1 Department of Biology and Environmental Science, Linnæus University, SE-39182 Kalmar, Sweden; 2 Department of Geobiology, Geoscience Centre Göttingen of the Georg-August University, Goldschmidtstrasse 3, 37077 Göttingen, Germany; 3 Department of Geological Sciences, Stockholm University, SE-106 91 Stockholm, Sweden; 4 CSC-IT Center for Science, PO Box 405, FIN-02101 Esbo, Finland; 5 Laboratory for Isotope Geology, Swedish Museum of Natural History, PO Box 50 007, SE-10405 Stockholm, Sweden; 6 Department of palaeobiology and the Nordic Center for Earth Evolution (NordCEE), Swedish Museum of Natural History, PO Box 50 007, SE-10405 Stockholm, Sweden; 7 Department of Surfaces, Chemistry and Materials, SP Technical Research Institute of Sweden, PO Box 857, SE-50115 Borås, Sweden

## Abstract

Precipitation of exceptionally ^13^C-depleted authigenic carbonate is a result of, and thus a tracer for, sulphate-dependent anaerobic methane oxidation, particularly in marine sediments. Although these carbonates typically are less depleted in ^13^C than in the source methane, because of incorporation of C also from other sources, they are far more depleted in ^13^C (*δ*^13^C as light as −69‰ V-PDB) than in carbonates formed where no methane is involved. Here we show that oxidation of biogenic methane in carbon-poor deep groundwater in fractured granitoid rocks has resulted in fracture-wall precipitation of the most extremely ^13^C-depleted carbonates ever reported, δ^13^C down to −125‰ V-PDB. A microbial consortium of sulphate reducers and methane oxidizers has been involved, as revealed by biomarker signatures in the carbonates and S-isotope compositions of co-genetic sulphide. Methane formed at shallow depths has been oxidized at several hundred metres depth at the transition to a deep-seated sulphate-rich saline water. This process is so far an unrecognized terrestrial sink of methane.

## Abstract (web summary)

Precipitation of ^13^C-depleted authigenic carbonate is a tracer of sulphate-dependent anaerobic methane oxidation, particularly in marine sediments. Here, the authors present extremely ^13^C-depleted carbonates from deep granitoid rocks suggesting the presence of microbial sulphate reducers and methane oxidisers.

## Graphical abstract

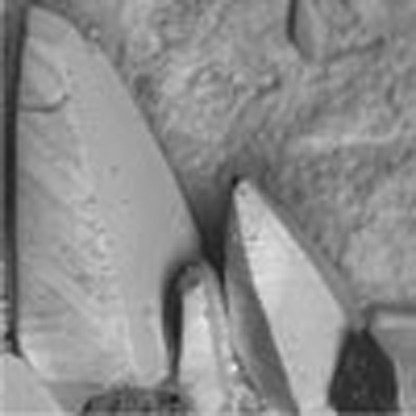

Although there are observations supporting anaerobic oxidation of methane (AOM) in deep groundwater in fractured granitic rock^1^,^2^,^3^,^4^, the vast majority of observations of this phenomenon are from marine seabed systems including fossil methane seeps^5^,^6^,^7^,^8^,^9^ and active methane seeps^10^,^11^,^12^,^13^. At marine methane seeps microbial AOM has been shown to occur with sulphate as an electron acceptor^14^, involving a defined, syntrophic two-membered bacterial consortium: anaerobic methane oxidizing (ANME) *archaea* and sulphate-reducing bacteria (SRB)^12^,^13^,^15^, although AOM solely by ANME has also been suggested^16^. The AOM occurs at the sulphate-methane transition zone (SMTZ), where sulphate-rich descending water mix with deeper-seated methane and SRB outcompete methanogens^12^,^13^,^15^. Here methane is oxidized by ANME via a proposed reversal CO_2_ reduction and, as a consequence, authigenic carbonates precipitate from the produced bicarbonate^9^. Because the methane generally has light carbon isotope values (^13^C/^12^C expressed as *δ*^13^C) of down to −50‰ when it is thermogenic and typically −60 to −110‰ when it is biogenic^17^, the authigenic carbonates are ^13^C-depleted with values as low as −69‰ (refs 5, 6, 7, 8, 10, 11, 12, 18).

In contrast to shallow marine sediments where sulphate is generally depleted with depth, numerous granitic shield areas worldwide host sulphate-rich water at great depth, in brines derived from a proposed marine source such as basinal brines from overlying sedimentary rock successions^19^,^20^,^21^. When the basement rock eventually has been re-exposed after erosion of the sedimentary successions, repeated infiltrations of surface water, including glacial melt water, marine water and meteoric water, are possible and controlled by factors such as location and topography. Fresh water (meteoric) therefore typically occupies the upper bedrock fracture volume, a mixture of brackish and glacial water is found at intermediate depth, and saline sulphate-rich water is generally restricted to great depth, such as described for the crystalline bedrock of southeastern Sweden in the Baltic Shield^22^,^23^. Owing to changing hydrochemical conditions during infiltration of occasionally carbon-rich water from the terrestrial environment above and increased alkalinity in response to microbial breakdown of organic matter and methane, carbonates (as a rule calcite) can become oversaturated and precipitate on the fracture walls. The stable carbon isotope composition of these calcites can thus be used to identify biological processes in the fractured upper crust, especially across depth-related hydrochemical boundaries, at which microbial communities thrive^14^. However, low-temperature calcite has only been sparsely used to reveal microbial processes in deep bedrock fractures because of its' rare, fine-grained and finely zoned nature, as well as because of challenges and costs of sampling representative calcite crystals deep in the Earth's crust^3^,^24^,^25^,^26^,^27^,^28^.

In the present study we have examined more than 40 cored boreholes drilled in one of the most extensively studied granitic bedrock sites in the world, the Laxemar area, Sweden, and sampled fine-grained-zoned calcite crystals throughout the upper 1,000 m of the bedrock. These calcites postdate fluid-inclusion-rich calcite formed at >50 °C (refs 25, 29) and have single-phased fluid inclusions suggesting, although few in numbers, formation at <50 °C (ref. 30). The stable isotopic composition of carbon and oxygen was determined in transects across the numerous growth zones present within the calcite crystals, in a total of more than 430 analyses by secondary ion mass spectrometry (SIMS; 10 μm wide, 1–2 μm deep spots). The sulphur isotope inventory, ^34^S/^32^S presented as *δ*^34^S, of co-genetic pyrite crystals were explored by similar SIMS analyses (*n*=101), which can reveal coeval SRB activity^31^. The organic inventory of the calcites and the groundwater chemistry are also explored. Thereby, the hydrochemical and biological evolution *in situ* in the fractures could be revealed in detail.

Here we report extremely ^13^C-depleted carbonates precipitated in fractures deep within granitoid rocks. The δ^13^C values are by far the lowest ever reported for carbonates (*δ*^13^C:−125‰) and are suggested to be related to microbial sulphate-dependent anaerobic oxidation of biogenic methane. Methane oxidation occurring in the energy-limited and carbon-poor groundwater systems deep within Earth's terrestrial landscape evidently results in a unique carbon isotope variability compared with other environments, such as the well-characterized sedimentary AOM settings.

## Results

### Extreme carbon isotope variation of calcite in rock fractures

**Figure 1:**
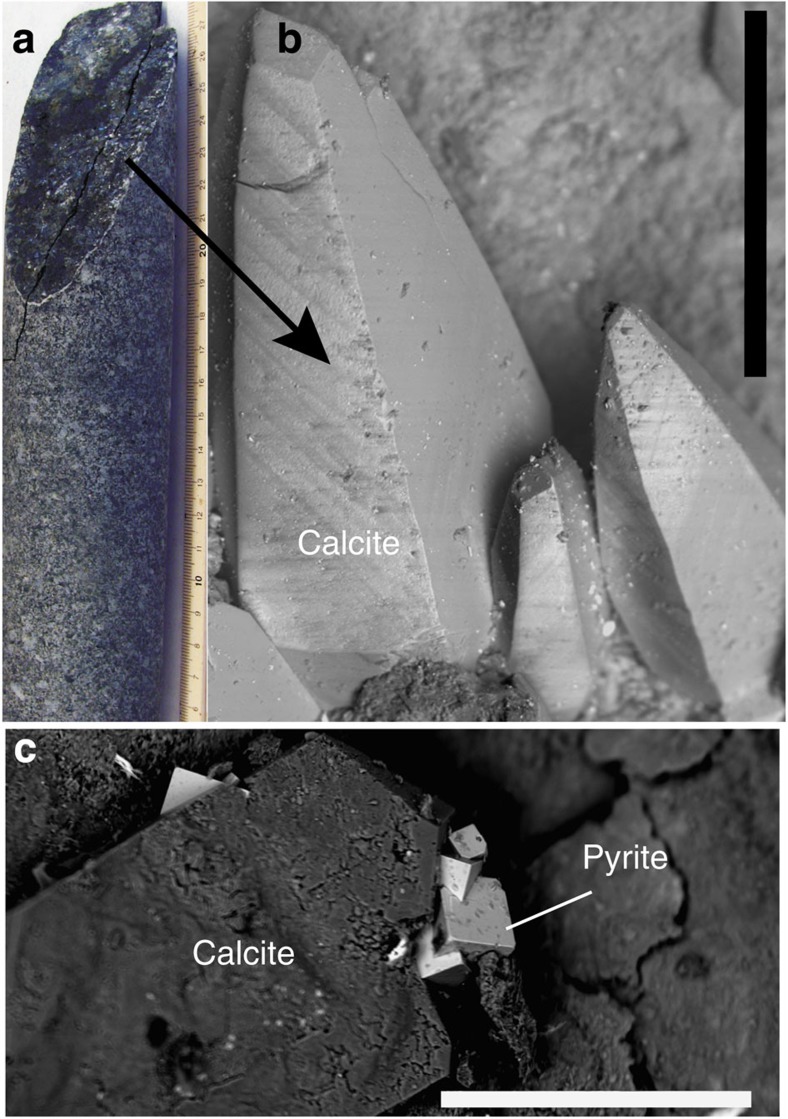
Fracture and mineral characteristics. (**a**) Drill core with an exposed fracture surface (scale in cm). (**b**,**c**) SEM images of crystals *in situ* on the fracture wall (scale bars, 500 μm). The calcite in **c** is formed via anaerobic oxidation of methane and is intergrown with pyrite formed in relation to bacterial sulphate reduction.

**Figure 2:**
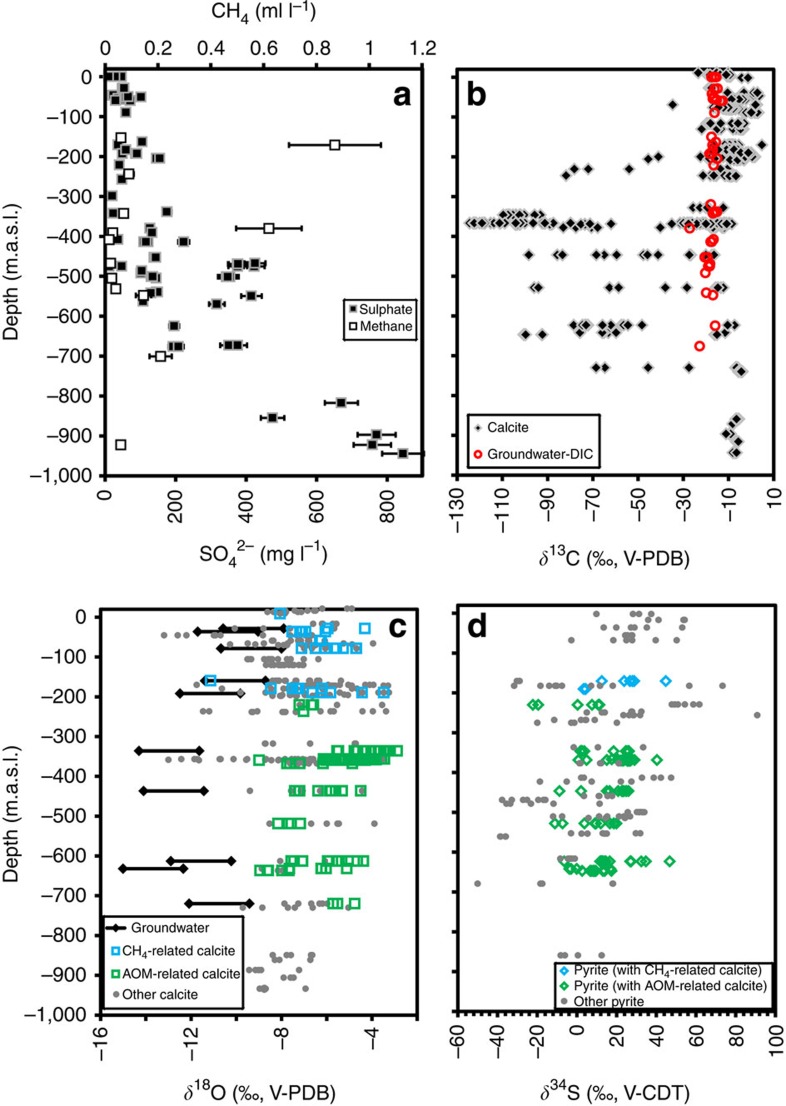
Depth-related variations of geochemical variables in the groundwater and in calcite. (**a**) Sulphate and methane concentrations^34^ in the groundwater. (**b**) *δ*^13^C_calcite_ and groundwater *δ*^13^C_DIC_. (**c**) *δ*^18^O_calcite_. Panel **c** includes a range for hypothetical calcite precipitated from the current groundwater at the same depth where the AOM- or methanogenesis-calcite coatings were collected. Equation 1,000 l*nα*(Calcite−H_2_0)=18.03(10^3 ^T^−1^)−32.42 (ref. 36) is used to calculate the fractionation factor (*α*) between oxygen in water and calcite at borehole water temperatures of 5–20 °C (hence the range). (**d**) *δ*^34^S values of pyrite in paragenesis with AOM-related crystals, together with pyrite in paragenesis with methanogenesis-related calcite and pyrite without any indicated methane relation^31^. For the stable isotope analyses, the symbol sizes are larger than the analytical uncertainties. Groundwater data are listed in Supplementary Table 2 and full stable carbon, oxygen and sulphur isotope data for calcite and pyrite in Supplementary Tables 1 and 3.

**Figure 3:**
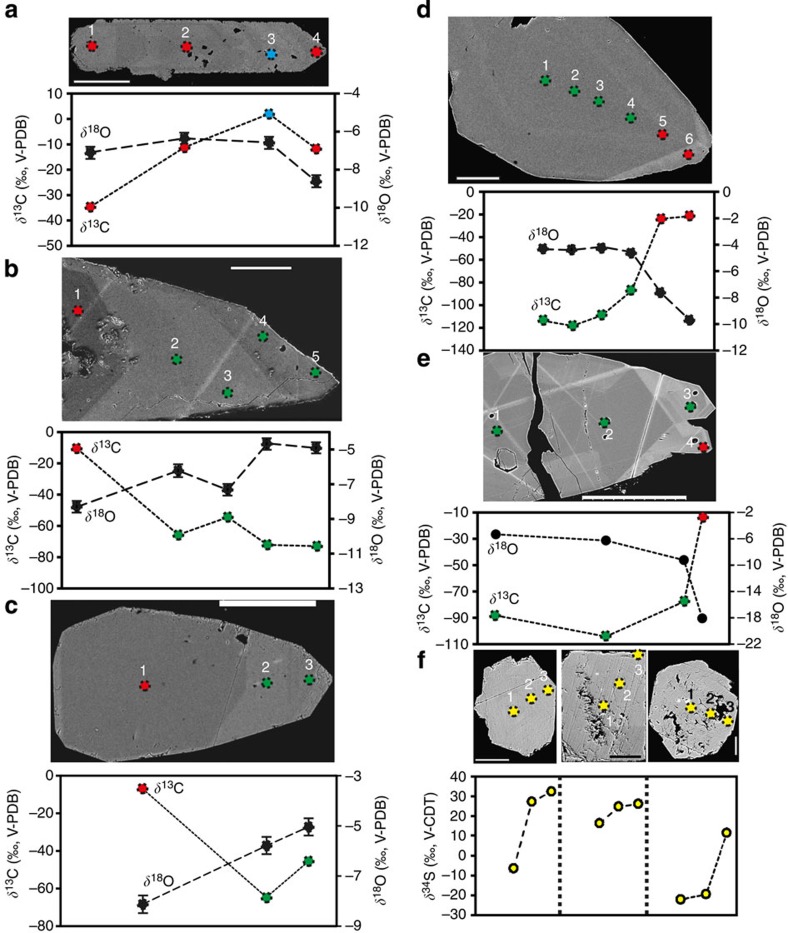
Variation of stable isotope composition within different calcite and pyrite crystals. Transects of SIMS analyses are shown in back-scattered SEM images above, and isotopic compositions corresponding to these analyses below. Growth direction of calcite is from left to right. (**a**) Calcite with episodic methanogenesis-related signature (positive *δ*^13^C, blue symbol). This is the dominant appearance of methanogenesis-related calcite, related to *δ*^18^O with marine-influenced signature followed by lighter *δ*^13^C and *δ*^18^O. (**b–e**) AOM-related calcite (green symbols) with typical associated increase in *δ*^18^O values from the earlier growth zone (indicative of increased marine influence). (**d,e**) AOM-related calcite succeeded by calcite with significantly heavier *δ*^13^C and lighter *δ*^18^O (fresh water, with large glacial component, especially in **e**). (**f**) *δ*^34^S evolution with growth from core to rim in pyrite from three different fractures. The symbol sizes are generally larger than the analytical uncertainties (except for *δ*^18^O, where error bars are shown). Scale bars (**a**) 300 μm, (**b**) 200 μm, (**c**) 200 μm, (**d**) 100 μm, (**e**) 200 μm, (**f**) 50 μm.

Within the individual calcite crystals (Fig. 1) there is large *δ*^13^C variation, of up to almost 110‰ between different growth zones, thus revealing large temporal variation of the processes in the fractures (Figs 2b and 3, Supplementary Table 1, location in Supplementary Fig. 1). The range of the *δ*^13^C values depends on the depth at which the calcites were precipitated and reside and, therefore, mark different biological processes at different depths within the fracture network (Fig. 2b). In the upper 200 m several calcites have zones with positive *δ*^13^C values (for example, in Fig. 3a). Such ^13^C-enriched carbonate pools develop where methanogenesis occurs^32^,^33^, leading to a ^12^C-rich methane and ^13^C-rich residual CO_2_ from which calcite formed. Although measurements of methane are relatively scarce in this area and the concentrations are generally low (<0.2 ml l^−1^), anomalies with elevated concentrations (>0.6 ml l^−1^, related to waters with abundant cultivatable methanogens and high concentrations of dissolved organic carbon, DOC^34^), are indeed restricted to shallower depths than the sulphate-rich saline waters (Fig. 2a, Supplementary Table 2). The organic input to the system in the form of descending DOC, which fuels the methanogenesis, evidently has a surficial origin.

Extreme ^13^C depletion of −125 to −50‰ was observed in calcites between 200 and 730 m. This ^13^C depletion is so strong that it cannot originate from any other source compound than methane. For methane to be incorporated in calcite, it first has to be oxidized, in this particular setting by anaerobic mechanisms because of consistently reducing conditions (absence of O_2_; Eh ranges from −210 to −380 mV (ref. 22)), far below the redox transition at 20 m depth^35^. In addition, aerobic methane oxidation would have resulted in calcite dissolution rather than precipitation^36^. The oxidation of methane causes a decrease in ^13^C in the produced CO_2_, with fractionation (*α*_CH4–CO2_) commonly in the order of 1.009–1.024 (refs 17, 37, 38). The calcites with the lightest *δ*^13^C must therefore have been produced from a source methane with *δ*^13^C lighter than −100‰. Consequently, the C-isotope data suggest a dominantly biological origin of the source methane because thermogenic methane rarely exceed values lighter than −50‰ (ref. 17). Thirteen fractures from boreholes spread out over a 3 × 3 km area featured AOM-related calcite, which in seven fractures had lighter *δ*^13^C than −90‰ (four lighter than −100‰), marking that extraordinary ^13^C depletions are widespread in this environment and not single occurrences. All of these 13 fractures carried calcite with minimum *δ*^13^C values that were similar to or lighter than reported for AOM-related authigenic marine carbonate (−69‰ (ref. 5)). Minimum sulphur isotope values of pyrite (*δ*^34^S_pyrite_, full results in Supplementary Table 3) co-genetic with AOM calcite are mainly 22–33‰ lighter, and in the rare case 46.8‰ lighter, than the anticipated source sulphate (+25‰ (ref. 31)). This is evidence of activity of SRB, which preferentially use the lighter S isotope in their metabolism, with large related fractionation between sulphate and produced sulphide as a consequence^39^. Large fractionation of the sulphur isotopes characterizes AOM-related microbial sulphate reduction, particularly when methane concentrations are low, such as at SMTZ^40^. These findings are in accordance with previous observations of SRB-related pyrite in the deep fracture system at Laxemar^31^ (Fig. 2d) and at nearby Äspö^3^. In the latter case, pyrite was accompanied by ^13^C-depleted calcite (at lightest −46.5‰ V-PDB; Vienna Pee Dee Belemnite), although not specifically interpreted to be AOM-related^3^.

Other *δ*^13^C values than those related to methanogenesis (heavy) or AOM (light) within the crystals reflect a dominance of inorganic C (−10 to −3‰) observed at all depth, and dominance of C from microbial degradation of organic matter (down to −30±5, potentially reflecting mixing of different C sources, including a minor AOM part). The latter δ^13^C signatures appear down to −730 m, which thus represent a depth limit for the descent of DOC from the terrestrial ecosystem above.

### Organic inventory of the calcite crystals

**Figure 4:**
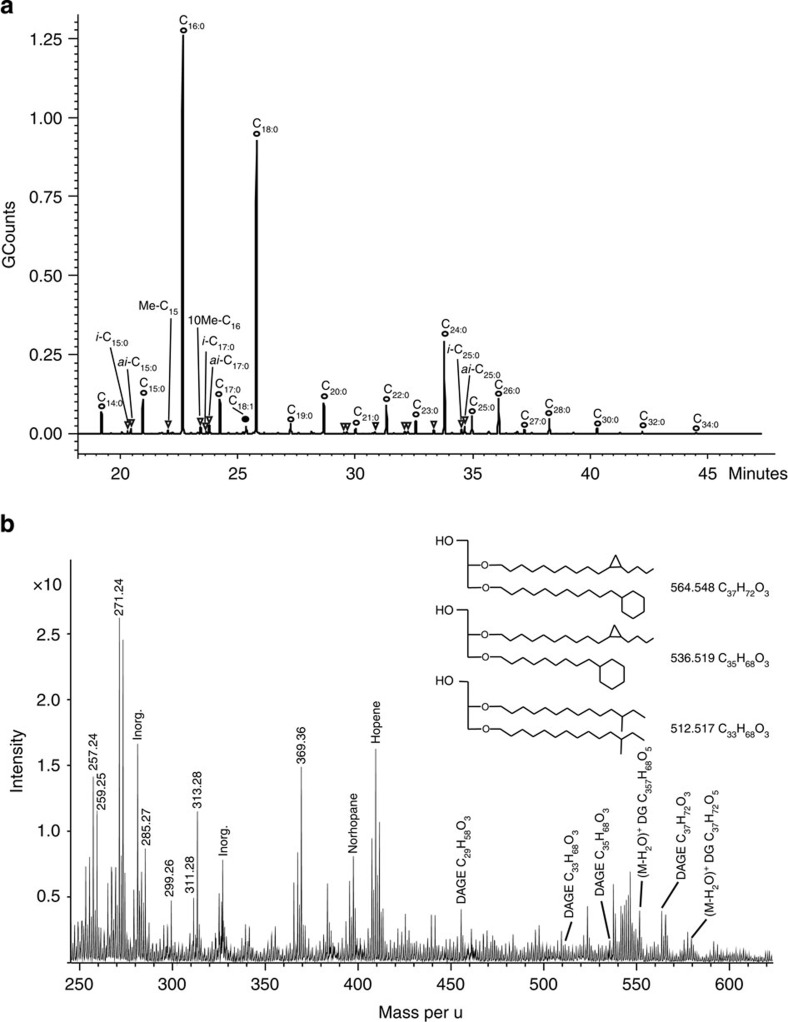
Organic compounds detected in a calcite leachate (210 mg) from KLX03−623 m. (**a**) GC–MS. Fatty acids (FA) observed with GC–MS can be separated into short-chain FA to a bacterial contribution (C_14_ to C_19_), with *i*- and *ai*-C_15_; *10Me*-C_16_; *i*- and *ai*-C_17_ being very common in SRB, and long-chain FA (C_20_ to C_34_) that may represent a diagenetic signature of high plants. (**b**) ToF-SIMS spectrum revealing the presence of hopanoids, DAGE and ester-bound diacylglycerols (DGs). Exact masses and characteristic fragments observed are given in Supplementary Table 4.

In addition to the diagnostic ^13^C-depleted carbonate, the presence of typically ^13^C-depleted specific lipid biomarker signatures of ANME and its SRB partner frequently serve as diagnostic tracers for active or fossil AOM in marine settings^14^,^18^,^41^. In the tiny calcite crystals from bedrock fractures studied here, it is impossible to determine *δ*^13^C of specific organic compounds. Nevertheless, our coupled gas chromatograph mass spectrometer (GC/MS) and Time-of-Flight (ToF)-SIMS analyses revealed signatures of methylated *anteiso*- (*ai*) and *iso*- (*i*) fatty acids (*ai*-C_15:0_, *10Me*-C_16:0_, *i*- and *ai*-C_17:0_, Fig. 4), hopanes (norhopane and hopene) and several non-isoprenoidal dialkyl glycerol diethers with branched alkyl, cyclohexyl or cyclopropyl units (DAGE, Fig. 4, Supplementary Table 4), in the AOM-related calcites. These organic molecules clearly originate from *in situ* microbial activity because they are mostly SRB-specific, and in the case of DAGEs, and *ai*-C_15:0_ highly AOM-specific^8^,^9^,^18^. This suggests that a similar two-membered ANME-SRB microbial consortium as described at other AOM sites^13^,^18^ operated here as well. The organic material within the calcites generally clusters along intracrystal grain boundaries (the borders between different growth zones), as shown by ToF-SIMS analyses of crystal interiors. Raman spectroscopy and ToF-SIMS revealed that there is no abundant organic material in the calcite matrix apart from along these grain boundaries. The GC/MS and ToF-SIMS analyses of calcite leachates (from up to 220 mg large calcite samples), which represent accumulated organic material from grain boundaries of several crystals showed more pronounced signatures of organic compounds compared with spot analyses of the crystals. Raman spectroscopy and scanning electron microscopy (SEM) investigations showed, however, that fine-grained clay minerals and pyrite dominate along the grain boundaries (Supplementary Fig. 2, Supplementary Note 1 and Supplementary Table 5). Numerous observations from crystalline rock fractures and vesicles elsewhere report complete mineralization of microbial structures to, for example, clay minerals, embedded in calcite^42^,^43^,^44^. Significant, but not complete, fossilization of the microorganisms in biofilms at the grain boundaries is therefore proposed on the basis of the observations of the interiors of the AOM crystals.

### General mechanisms of methane formation and oxidation

Taken together, the *δ*^13^C data of the calcites suggest a mechanism whereby methane is formed in shallow fractures (Fig. 2a,b), transported downwards and oxidized and precipitated as calcite at the border to the sulphate-rich, methane-poor saline water deep in the crust at 200–730 m. The oxygen isotope composition (^18^O/^16^O expressed as *δ*^18^O) of the calcites can be used to link the calcites to different climatic/hydrological events through comparison with the *δ*^18^O of the infiltrating waters, which in the studied setting is approximately −21‰ V-SMOW for glacial water, −6 to 0‰ for the variety of transgressed marine and/or brackish waters (ocean-type water at *c.* 0‰, Holocene brackish Baltic Sea waters at −5.9 to −4.7‰), −10‰ for meteoric and c. −9‰ for the deep sulphate-rich brine at >500 m depth^45^. Although mixing of these waters have resulted in mixing of the *δ*^18^O values, generally one of the water types dominates^45^. Because the *δ*^18^O values of the AOM- and methanogenesis-related calcites are generally similar and constant at −6±2‰ PDB with depth (Fig. 2b), these two types of calcites can be causally and temporally linked to the infiltration of a similar type of groundwater. On the basis of the *δ*^18^O values and consideration of the fractionation occurring when calcite precipitates, this water was dominantly brackish-marine (*δ*^18^O values in line with brackish/marine waters during Holocene and the Eemian interstadial, *cf.*^22^,^46^, and heavier than all other potential source water types). The AOM- and methanogenesis-related calcite generally do not make up the whole crystals but can be both preceded and succeeded by calcite with distinctly different *δ*^13^C- and *δ*^18^O-signatures (Fig. 3b–e). This reflects that methanogenesis and AOM have been initiated but also terminated in response to changing hydrochemical conditions in the fracture system. The AOM-related calcite growth is frequently accompanied by relative enrichment in ^18^O in the calcites, indicative of increased proportion of marine water relative to fresh waters (Fig. 3b,c), further supported by brackish-marine salinities (2.4 wt.% NaCl) estimated in the only AOM sample with measurable fluid inclusions (at −642 m, Supplementary Table 6, Supplementary Note 2, Supplementary Figs 3 and 4). This is in line with the sequence of infiltration of surficial water during the repeated Pleistocene deglaciation cycles in this area, involving high hydraulic heads that pressed glacial melt water several hundred metres into the fracture network and subsequent marine transgressions over the depressed land masses^22^. During the marine transgression, dense marine (brackish) water was brought on top of light fresh water in the fractures. In such a system advection can occur, under ideal conditions, with a velocity of up to the order of 1 cm s^−1^ (see Supplementary Note 3), corresponding to a travel time of about a single day for a vertical downward distance of 500 m. In contrast to methane diffusion, which is too slow (Supplementary Note 3), advective transport, particularly favourable after a glaciation but certainly possible also in other situations, is thus fully capable of carrying dissolved methane deep into the crust, down to the deep sulphate-rich brines, within a short period of time.

Marine waters descending through organic-rich sediments will have delivered to the superficial fractures dissolved organic matter that favour methanogenesis but also dissolved sulphate that will lead to methane oxidation. In near-surface fractures, however, pyrite crystals with extremely heavy *δ*^34^S values of up to more than +90‰ V-CDT (Vienna Canyon Diablo Troilite) exist^31^. This is evidence for extensive sulphate consumption by SRB because values as heavy as these can have been formed only where the dissolved sulphate pool was nearly exhausted in the fracture. Consequently, because sulphate was exhausted, methane concentrations were allowed to build up and descend downwards.

### Dominantly pre-Holocene methane oxidation

The Holocene post-glacial marine transgression generally did not reach as far inland as the investigated boreholes. Hence, the groundwater captured by these is still made up of a significant portion of glacial meltwater on top of the deep saline water. This C-poor Holocene glacial meltwater mixed with and/or replaced an older marine water, to which AOM is possibly related. Therefore, ongoing AOM is not believed to be significant at this site. This scenario is supported by (1) AOM-related and methanogenesis-related calcite being succeeded by calcite without AOM *δ*^13^C signature of fresh water type (dominantly glacial or meteoric water replacing the marine/saline water, Fig. 3d,e), (2) the overall heavier *δ*^18^O of the AOM- and methanogenesis-related calcites (dominantly marine) than those expected for calcite precipitated from the current groundwater with a large glacial component at great depth and a large meteoric component at shallow depth (Fig. 2c) and (3) limited variation in *δ*^13^C_DIC_ values in the current groundwater compared with the calcites. This supports dominant formation of the AOM calcites in a groundwater system predating the latest (Weichselian) glaciation but within the last 10 Ma when the Paleozoic cover had been eroded away^47^, allowing the border to the deep sulphate-rich water to be depressed to depths of several hundred meteres where AOM calcites formed. A minor number of overlapping calculated *δ*^18^O values of shallow groundwater and calcite (Fig. 2c) indicate potential but limited methanogenesis-related (and minor deeper AOM-related) calcite formation from the current DOC-rich meteoric waters at these depths, in accordance with the observed scattered enhanced methane concentrations (Fig. 2a) and presence of methanogens and SRB^34^.

## Discussion

**Figure 5:**
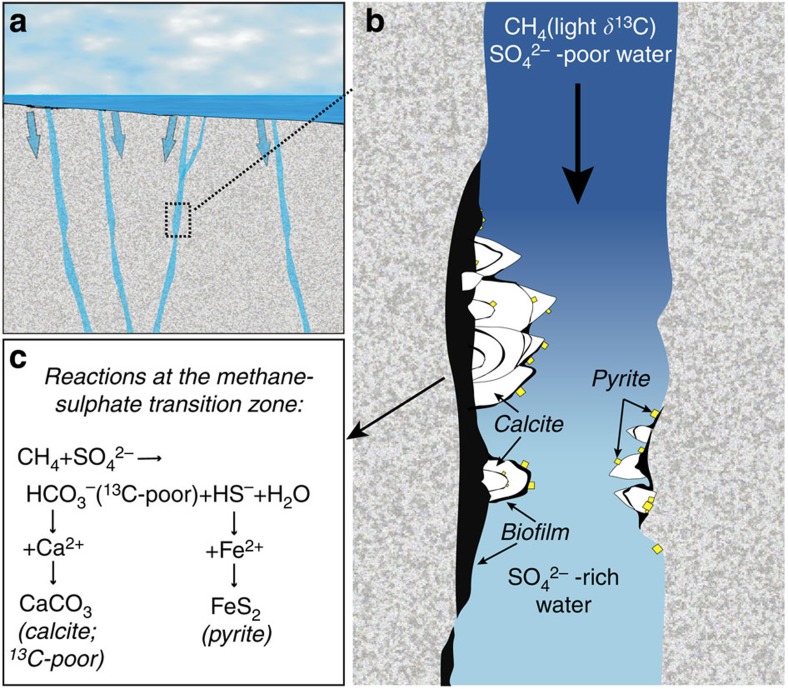
Schematic images of the processes in the fractures. (**a**) An overview including typically near-vertical to vertical water-conducting fractures through which marine waters descended (width of view c. 1 km). (**b**) Conditions and (**c**) reactions occurring locally in open fractures (width of view in **b** is c. 700–800 μm). Sulphate-poor descending fluids containing the methane mix with a deeper sulphate-rich, bicarbonate-poor water. At this transition AOM occurs, involving bacterial sulphate reduction promoting pyrite precipitation and increased alkalinity triggering calcite formation. AOM occurs preferentially in microbial communities (black, degraded over time) at the fracture walls, and the incorporation of carbon into calcite is therefore dominated by products of the local AOM process, as shown by both the *δ*^13^C values, and by the closed system conditions of the sulphate reduction (evidenced by the *δ*^34^S evolution within the pyrites).

Our study shows that AOM-related calcite precipitation was established at the border between a descending sulphate-exhausted water, carrying dissolved methane, and a deeper sulphate-rich old saline water. The processes are outlined in Fig. 5 and include reduction of sulphate to ^34^S-depleted sulphide and oxidation of ^13^C-depleted biogenic methane to ^13^C-depleted bicarbonate by a syntrophic consortium of ANME and SRB. These reactions cause an increased alkalinity invoking precipitation of ^13^C-depleted calcite on the fracture wall. This calcite formed from reaction of the produced bicarbonate with the abundant dissolved Ca^2+^ in the deep saline water (up to 740 mg l^−1^ (ref. 24)). The most likely explanation why the calcites are considerably more ^13^C-depleted than the numerous marine AOM calcites observed worldwide is that the latter form in DOC- and DIC-rich sediment porewaters and thus more readily incorporate carbonate from other carbon sources than oxidized methane^9^,^48^. In strong contrast, the deep groundwater in crystalline rocks carries low concentrations of DIC and DOC (in Laxemar, each less than 2 mg l^−1^ compared with maximum values of 330 and 21 mg l^−1^, respectively, in the upper 200 m), indicating that methane has been an almost exclusive carbon source for the ^13^C-depleted calcites. The low concentrations of DOC in the deep groundwater were likely also partly refractory to microbial degradation in similarity with the limited availability and poor reactivity of organic substrates in deep-sea sediments^49^. Pyrite (depleted in ^34^S compared with sulphate) is formed by reaction of the SRB-produced dissolved sulphide and Fe^2+^ (present at 0.1–0.8 mg l^−1^ (ref. 24)). The large range in *δ*^34^S_pyrite_ of 68.9‰ (−22.1 to +46.8‰, for example, in Fig. 3f) and frequent progressively heavier *δ*^34^S with growth clearly reflect bacterial sulphate reduction in a system where the reduction rate has exceeded the supply of sulphate by advection and diffusion^50^. Increase in *δ*^34^S values by up to almost 40‰ over a crystal growth distance of 50 μm (Fig. 3f) strongly suggests very local microscale isotope systematics at the fracture wall, in spatial relation to microbial communities^31^. Similarly, the contrasting *δ*^13^C values in the calcites compared with those in the bulk fracture water (Fig. 3b) support local incorporation of ^13^C-depleted bicarbonate formed by AOM at the fracture wall. However, the generally irregular *δ*^13^C evolution within these calcites rules out similar closed system evolution of the carbon system as for the sulphur system. The irregular *δ*^13^C variation of up to 45‰ in the AOM-related zone within the calcite crystals instead reflects either (1) local variability of incorporation of carbonate into calcite from other carbon sources than methane and/or (2) substantial variation of the *δ*^13^C values of the oxidized biogenic methane as a result of methanogenesis during substrate-limited conditions^51^ occurring in the deep biosphere, or because of other factors that influence the kinetic carbon isotope effect during methanogenesis, including sulphate availability (*cf.* ref. 52), substrate utilized and temperature^17^,^51^. As the *δ*^13^C value of the oxidized methane is unknown, we cannot exclude that the source methane of the <−100‰ calcites has been extremely depleted in ^13^C. It has recently been demonstrated that low sulphate concentrations (<50 mg l^−1^) can lead to microbially mediated carbon isotope equilibration between methane and carbon dioxide resulting in some degree of reversibility of the methane oxidation (that is, back-flux)^53^,^54^. This causes progressively decreased *δ*^13^C values of the residual methane. An extraordinary ^13^C-depleted methane, affected by partial and reversible oxidation in a sulphate-limited system, such as in most of the current shallow and intermediate waters (Fig. 2a), can therefore indeed have been the source of the extremely ^13^C-depleted calcites.

The setting described here is not unique for the Baltic Shield, but is found on all continents in a variety of regions. Therefore, the identified methane-consumption mechanism in bedrock fractures and similar ^13^C-depleted carbonates can occur worldwide. Our finding of AOM at a transition between biogenic methane formed at shallow depth and a deeper sulphate-rich saline water is, however, completely reverse to the SMTZ observed in typical well-characterized sedimentary AOM settings and at another well-characterized crystalline rock site (Olkiluoto, Finland)^1^,^2^, where deep-seated methane instead intersects with sulphate from superficial layers. Deep sulphate-bearing brine incursions that fuel AOM below the SMTZ have, however, been reported from a few marine sediments as well^55^. Although hydrochemical data are scarce from great depth in crystalline rocks, deep sulphate-rich waters, capable of ultimately producing the reversed SMTZ as described here, have been reported elsewhere^19^,^20^,^56^,^57^, offering opportunities for similar biogeochemical processes on a global scale. Although SRB- and methanogenesis-related isotopic signatures have been reported from groundwater and minerals in fractured crystalline rock in for example Sweden, Finland and USA^3^,^26^,^31^,^33^,^58^,^59^,^60^, and a few scattered possible AOM indications of calcite (bulk samples) from Sweden exist^3^,^59^,^61^, more detailed and comprehensive *in situ* studies of low-temperature calcite are needed to better constrain the extent of past and present AOM deep in crystalline rocks globally. The episodic nature of the processes indicates dependency of certain conditions for initiation of AOM that may vary from site to site.

In conclusion, our study reveals a previously unknown methane–sulphate transition zone with consumption of biogenic methane at the border to sulphate-rich deep saline water at great depth in crystalline rocks. The relatively energy-limited and C-poor environment at great depth within these granite fractures has resulted in utilization and almost exclusive incorporation of C from biogenic methane into the extremely ^13^C-depleted calcites precipitated on the fracture walls during AOM by ANME-SRB consortia. These findings greatly expand the observed range of carbon isotope variation in carbonates in natural environments, even when compared with sediment-hosted AOM occurrences, which previously were considered to host the most ^13^C-depleted carbonates globally. The conditions associated with methane oxidation deep within Earth's terrestrial landscape is evidently substantially different compared to methane oxidation in other environments.

## Methods

### SIMS

Calcite and pyrite crystals sampled from 18 drill cores were mounted in epoxy, polished to expose crystal cross-sections and examined using SEM (to trace zonations). Intracrystalline SIMS analysis (10 μm lateral beam dimension, 1–2 μm depth dimension) of carbon, oxygen and sulphur isotopes were performed on a Cameca IMS1280 ion microprobe. Analytical transects were made within several crystals from each sample. Settings follow those described in ref. 31, with some differences; O was measured on two Faraday cages (FC) at mass resolution 2,500, whereas C used a FC/EM combination, with mass resolution 2,500 on the ^12^C peak and 4,000 on the ^13^C peak to resolve it from ^12^C^1^H. Data were normalized using Brown Yule Marble (*δ*^18^O: 24.11±0.13‰ V-SMOW, converts to 6.55±0.13‰ V-PDB, *δ*^13^C: −2.28±0.08‰V-PDB, derived from three replicate bulk analyses, J. Craven, University of Edinburgh, personal communication) and Balmat pyrite (+16.515±0.005‰ V-CDT^62^). Precision was *δ*^18^O: ±0.3–0.4‰, *δ*^13^C: ±0.4–0.5‰ and *δ*^34^S: ±0.13‰. Potential matrix effects for SIMS analysis due to Mg and Fe substitutions in the calcites (*cf.* ref. 63) are negligible because of very low molar fractions of Mg and Fe (X_Mg_ and X_Fe_) in these and other low-temperature calcites in the area (up to only 0.002 and 0.004, respectively^24^,^64^). Significant influence of organic carbon was avoided in the SIMS analyses by careful spot placement to areas in the crystals without microfractures or inclusions, at a sufficient distance from grain boundaries where fine-grained clusters of other minerals and remnants of organic material may appear. The uncertainty associated with potential organic inclusions and matrix composition is therefore considered to be insignificant compared with the isotopic variations.

### ToF-SIMS and GC/MS

Extraction of calcites for analyses of organic compounds was carried out accordingly. Calcite powder was extracted with 2 ml of predistilled dichloromethane in a Teflon-capped glass vial (ultrasonication, 35 min, 50 °C). The supernatant was decanted after centrifuging. Extraction was repeated three times. After evaporation of the combined extracts and re-dissolution in pure dichloromethane, the solvents were dried with N_2_. Extracts were re-dissolved with 20 μl of n-Hexane and derivatized by adding 20 μl BSTFA (*N*,*O*-bis(trimethylsilyl)trifluoroacetamide)/pyridine and heated (40 °C, 1.5 h). Remnant calcite powder was decarbonized with BSTFA, TMCS (trimethylchlorosilane)/methanol and derivatized by heating (80 °C, 1.5 h). The lipid fraction was separated by mixing with hexane and decanting the supernate. Extraction was repeated three times. The samples were dried with N_2_, redissolved with 50 μl of n-Hexane and analysed with GC/MS and ToF-SIMS. One microlitre of each sample extract was analysed with a Varian CP-3,800 GC/1,200-quadrupole MS (settings in ref. 64). ToF-SIMS analyses of single calcite grains and organic extracts were conducted using a ToF-SIMS IV (ION-TOF GmbH, with liquid bismuth cluster ion source; settings in ref. 65). Compounds and corresponding characteristic fragments detected with ToF-SIMS and GC/MS within the AOM calcites are listed in Supplementary Table 4.

### Raman spectrometry

The Raman spectrometry analyses were performed on seven samples with a confocal laser Raman spectrometer (Horiba instrument LabRAM HR 800), equipped with a multichannel air-cooled (−70 °C) 1,024 × 256 pixel CCD (charge-coupled device) array detector. Acquisitions were obtained with a 1,800-line per mm grating. The excitation source was provided by a 514-nm Argon laser (Melles Griot 543) with a laser power at the sample surface of 8 mW. An Olympus BX41 microscope was coupled to the instrument. The laser beam was focused through a × 100 objective to obtain a spot size of ∼1 μm. The spectral resolution was ∼0.3 cm^−1^ per pixel. The accuracy of the instrument was controlled by repeated use of a silicon wafer calibration standard with a characteristic Raman line at 520.7 cm^−1^. The Raman spectra were achieved with the LabSpec 5 software. Results are briefly presented in Supplementary Note 1.

### Fluid inclusion microthermometry

Fluid inclusions were analysed in handpicked calcite crystals (0.5–1.5 mm in size) and in the epoxy-mounted crystals used for SIMS analysis following polishing of the mount to a 150-μm double-polished section. Microthermometric analyses of fluid inclusions were made with a Linkam THM 600 stage mounted on a Nikon microscope utilizing a × 40 long working-distance objective. The working range of the stage is from −196 to +600 °C (for details see ref. 66). The thermocouple readings were calibrated by means of SynFlinc synthetic fluid inclusions and well-defined natural inclusions in Alpine quartz. The reproducibility was ±0.1 °C for temperatures below 40 and ±0.5 °C for temperatures above 40 °C.

### Scanning electron microscopy

The zonation and inclusions of other minerals within polished hand-picked calcite crystals mounted in epoxy were examined using a Hitachi S-3,400 N SEM equipped with an integrated energy-dispersive spectroscopy system. The acceleration voltage was 20 kV, and the working distance 9.2 mm. During semiquantitative energy-dispersive spectroscopy analyses of other minerals within the calcites, oxide and element calibration standards (Smithsonian mineral standards) were used, linked to a cobalt drift standard (calibrated twice every hour) and a stable specimen current. Spot size was ∼5 μm.

### Hydrochemistry

Groundwater from water-conducting fractures were sampled in 3- to 10-m packed-off boreholes by SKB, and results were extracted from their database (SICADA). Only sections with <1% drilling water are used. Quality control of the analyses and details of methods are described in ref. 67.

## Author contributions

H.D. initiated and planned the project, carried out sampling, sample preparation, SEM-, SIMS- and ToF-SIMS analyses and wrote the manuscript together with M.E.A. C.H. carried out GC/MS and ToF-SIMS analyses and contributed to corresponding parts of the manuscript. C.B. and M.I. carried out fluid inclusion microthermometry and Raman spectroscopy and contributed to corresponding parts of the manuscript. J.A. carried out physical modelling and wrote to Supplementary Note 3. M.W. carried out SIMS analysis. S.S. and P.S. carried out ToF-SIMS analyses.

## Additional information

**How to cite this article**: Drake, H. *et al*. Extreme ^13^C depletion of carbonates formed during oxidation of biogenic methane in fractured granite. *Nat. Commun.* 6:7020 doi: 10.1038/ncomms8020 (2015).

## Supplementary Material

Supplementary Information Supplementary Figures 1-4, Supplementary Tables 1-6, Supplementary Notes 1-3 and Supplementary References
