# Supplementary material for: Extreme 13C depletion of carbonates formed during oxidation of biogenic methane in fractured granite
Source: Nat Commun. 2015 May 7;6:7020. doi: 10.1038/ncomms8020 (PMC4432592; doi:10.1038/ncomms8020)
Supplement: Supplementary Information — Supplementary Figures 1-4, Supplementary Tables 1-6, Supplementary Notes 1-3 and Supplementary References [file ncomms8020-s1.pdf]

Supplementary Figures

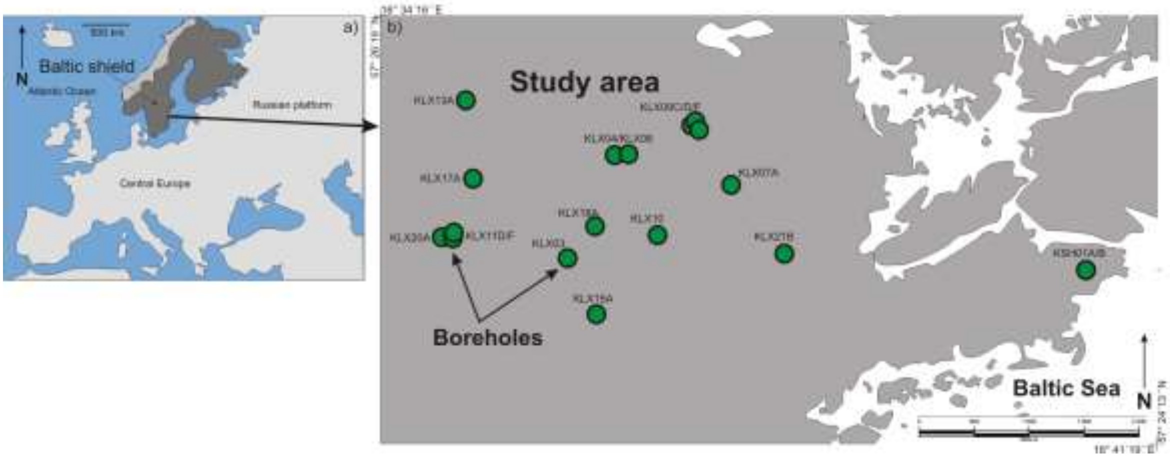

Supplementary Fig. 1. Location of the study area on the east coast of Sweden on the Baltic Shield, Northern Europe (a) and locations of the boreholes sampled for mineral precipitates in the study area (b).

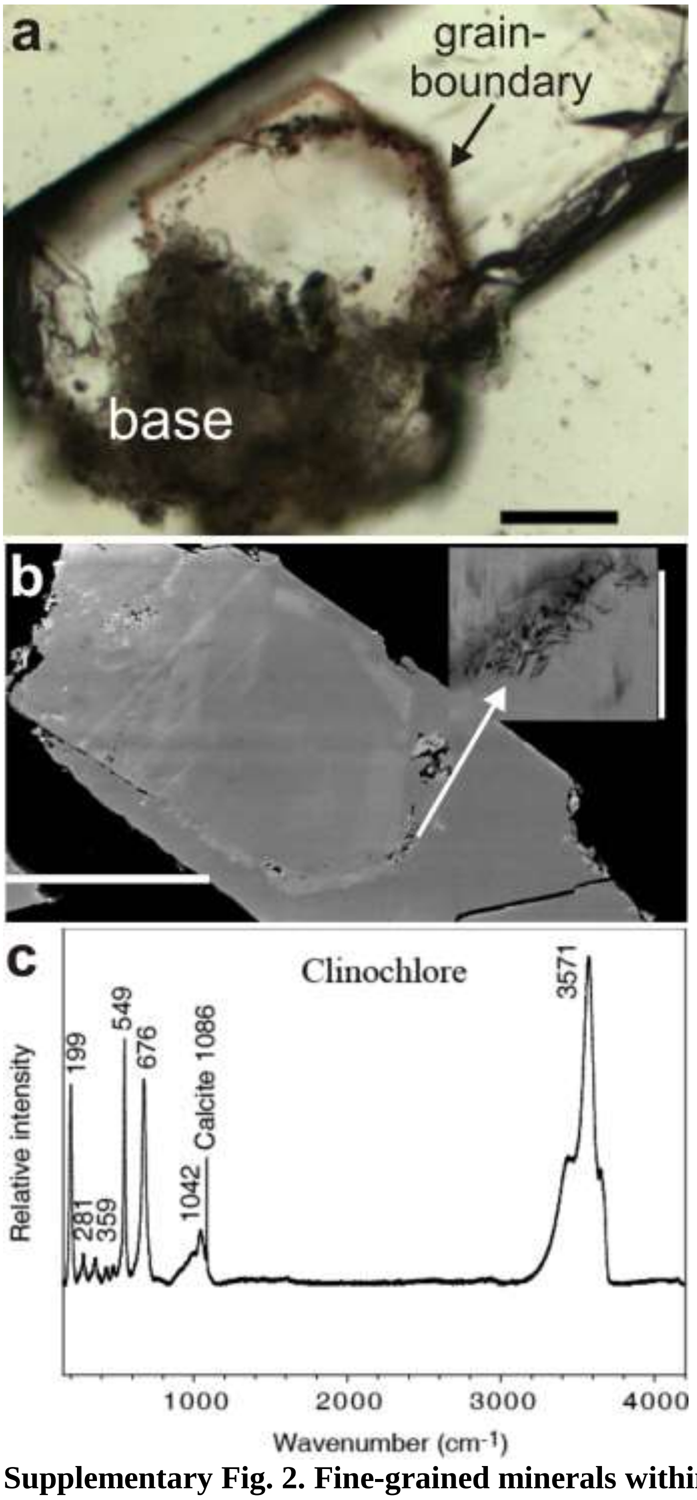

Supplementary Fig. 2. Fine-grained minerals within calcite. (a) Microphotograph showing one of the polished calcite crystals analysed in this study (sample KSH01A:-200 m, mounted in epoxy and polished). Clusters of fine-grained minerals are found at the base of the crystals (at the wall-rock contact) and within the crystal, along grain-boundaries between growth-zones. Scale bar is 50 µm. (b) Back-scattered SEM image of calcite (KSH01B:-39 m) with visible zonation, marked by intensity differences and clusters of fine-grained minerals (close up shows chlorite-dominated mixed layer-clay at a grain-boundary (composition in Supplementary Table 5). Scale bar is 300 µm (inset scale bar is 100 µm). (c) Raman spectrum from the grain-boundary in (a). The minerals are identified as calcite and clinochlore after comparison with reference spectra from the RRUFF project[6]. Bands from the calcite are indicated by Calcite + band.

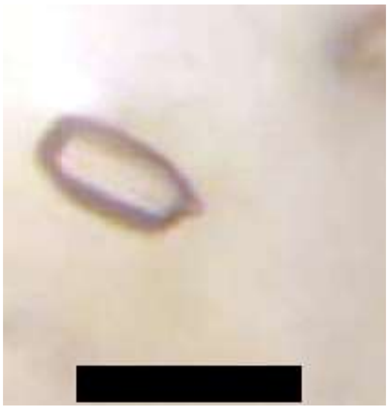

Supplementary Fig. 3. Fluid inclusion. Typical appearance of an all-liquid aqueous inclusion. Scale bar is 10 µm.

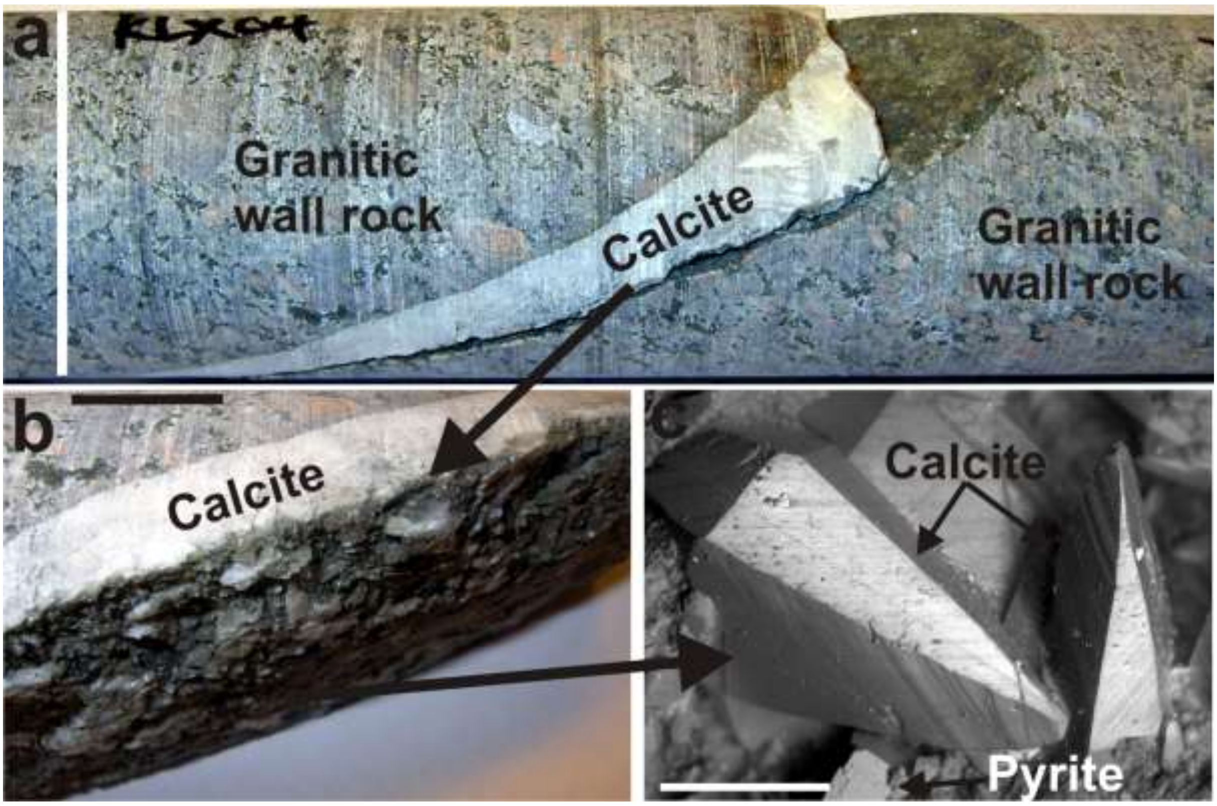

Supplementary Fig. 4. Relation between the calcite sampled in the present study and earlier formed fluid inclusion-rich calcite (Paleozoic). (**a**) Photograph of a calcite-vein in borehole KLX04: -642 m depth. This calcite has a stable isotope composition, δ^{18}O:-15.4‰ and δ^{13}C:-13.7‰, typical of Paleozoic calcite (dated to 450-400 Ma) formed from brine-type fluids (15-25 eq. wt.% $CaCl_2$) at temperatures above 50°C, shown by abundant fluid-inclusions analysed previously within Paleozoic calcites in this area[10-12]. The fracture has been re-activated at a later event along one of the wall rock contacts. Scale bar is 5 cm. (**b**) Photograph of the coating on the fracture wall of the open fracture post-dating the Paleozoic calcite vein. Scale bar is 2 cm. (**c**) Back-scattered SEM-image of euhedral calcite and pyrite precipitated on the fracture surface of the open fracture. The calcite mainly has AOM-related δ^{13}C-signature (-75.7 to -59.7‰) with significantly heavier δ^{18}O (-7.9 to -5.4‰) than the Paleozoic calcite. Scale bar is 500 µm.

Supplementary Tables

Supplementary Table 1. SIMS analyses of calcite

Borehole	Depth (m.a.s.l.)	Crystal	$\delta^{13}C$ (‰V-PDB)	± ‰	$\delta^{18}O$ (‰V-PDB)	± ‰	Borhole coordinates N	E
KLX17A	-346	1	-101.9	0.4	-3.8	0.3	57° 25´36´´	16° 35´11´´
KLX17A	-346	1	-104.1	0.4	-4.5	0.4	57° 25´36´´	16° 35´11´´
KLX17A	-346	1	-93.2	0.4	-5.0	0.3	57° 25´36´´	16° 35´11´´
KLX17A	-346	2	-106.6	0.4	-4.0	0.4	57° 25´36´´	16° 35´11´´
KLX17A	-346	2	-107.4	0.4	-3.7	0.3	57° 25´36´´	16° 35´11´´
KLX17A	-346	2	-100.4	0.4	-4.7	0.4	57° 25´36´´	16° 35´11´´
KLX17A	-346	3	-107.4	0.4	-3.5	0.4	57° 25´36´´	16° 35´11´´
KLX17A	-346	3	-107.5	0.4	-4.3	0.4	57° 25´36´´	16° 35´11´´
KLX17A	-346	3	-95.8	0.4	-3.2	0.4	57° 25´36´´	16° 35´11´´
KLX17A	-346	4	-108.3	0.5	-5.8	0.3	57° 25´36´´	16° 35´11´´
KLX17A	-346	4	-104.0	0.5	-4.6	0.3	57° 25´36´´	16° 35´11´´
KLX17A	-346	4	-105.3	0.5	-5.8	0.3	57° 25´36´´	16° 35´11´´
KLX17A	-346	4	-107.5	0.5	-4.9	0.4	57° 25´36´´	16° 35´11´´
KLX17A	-346	5	-109.6	0.5	-5.0	0.3	57° 25´36´´	16° 35´11´´
KLX17A	-346	5	-102.4	0.5	-5.1	0.3	57° 25´36´´	16° 35´11´´
KLX04	-529	1	-96.0	0.4	-7.4	0.3	57° 25´38´´	16° 36´26´´
KLX04	-529	1	-13.4	0.5	-7.0	0.3	57° 25´38´´	16° 36´26´´
KLX04	-529	2	-94.1	0.4	-8.4	0.4	57° 25´38´´	16° 36´26´´
KLX04	-529	2	-15.7	0.5	-6.3	0.4	57° 25´38´´	16° 36´26´´
KLX04	-529	2	-12.2	0.5	-5.7	0.4	57° 25´38´´	16° 36´26´´
KLX04	-529	2	-37.9	0.5	-4.2	0.4	57° 25´38´´	16° 36´26´´
KLX04	-529	3	-62.6	0.4	-7.9	0.4	57° 25´38´´	16° 36´26´´
KLX04	-529	3	-58.6	0.4	-7.8	0.3	57° 25´38´´	16° 36´26´´
KLX04	-529	3	-14.6	0.5	-8.8	0.3	57° 25´38´´	16° 36´26´´
KLX04	-529	3	-28.4	0.5	-5.8	0.3	57° 25´38´´	16° 36´26´´
KLX13A	-369	1	-9.0	0.5	-7.4	0.4	57° 25´51´´	16° 35´1´´
KLX13A	-369	1	-8.5	0.5	n.a.		57° 25´51´´	16° 35´1´´
KLX13A	-369	1	-24.8	0.5	n.a.		57° 25´51´´	16° 35´1´´
KLX13A	-369	2	-8.5	0.5	-6.2	0.4	57° 25´51´´	16° 35´1´´
KLX13A	-369	2	-71.3	0.4	-4.4	0.4	57° 25´51´´	16° 35´1´´
KLX13A	-369	2	-17.9	0.5	-9.8	0.4	57° 25´51´´	16° 35´1´´
KLX13A	-369	3	-35.1	0.7	-9.0	0.4	57° 25´51´´	16° 35´1´´
KLX13A	-369	3	-22.6	0.5	-8.4	0.4	57° 25´51´´	16° 35´1´´
KLX13A	-369	3	-13.0	0.5	-6.1	0.5	57° 25´51´´	16° 35´1´´
KLX13A	-369	3	-24.1	0.5	-6.1	0.4	57° 25´51´´	16° 35´1´´
KLX13A	-369	4	-10.9	0.5	-7.2	0.4	57° 25´51´´	16° 35´1´´
KLX13A	-369	4	-27.7	0.5	-4.7	0.4	57° 25´51´´	16° 35´1´´
KLX13A	-369	4	-20.0	0.5	-7.7	0.5	57° 25´51´´	16° 35´1´´
KLX13A	-369	5	-11.1	0.5	-7.2	0.4	57° 25´51´´	16° 35´1´´
KLX13A	-369	5	-61.9	0.5	-4.1	0.5	57° 25´51´´	16° 35´1´´
KLX13A	-369	5	-10.8	0.5	-13.2	0.4	57° 25´51´´	16° 35´1´´
KLX03	-623	1	-7.3	0.5	-6.4	0.4	57° 25´1´´	16° 35´52´´
KLX03	-623	1	-57.4	0.4	-7.3	0.4	57° 25´1´´	16° 35´52´´
KLX03	-623	1	-78.5	0.4	-5.6	0.4	57° 25´1´´	16° 35´52´´
KLX03	-623	1	-73.6	0.5	-6.2	0.4	57° 25´1´´	16° 35´52´´
KLX03	-623	2	-48.3	0.5	-7.8	0.3	57° 25´1´´	16° 35´52´´
KLX03	-623	2	-56.4	0.4	-7.7	0.3	57° 25´1´´	16° 35´52´´
KLX03	-623	2	-74.6	0.4	-5.8	0.4	57° 25´1´´	16° 35´52´´
KLX03	-623	2	-62.0	0.4	-5.3	0.4	57° 25´1´´	16° 35´52´´

KLX03	-623	3	-10.4	0.5	-8.3	0.4	57° 25´1´´	16° 35´52´´
KLX03	-623	3	-65.7	0.4	-6.2	0.4	57° 25´1´´	16° 35´52´´
KLX03	-623	3	-54.4	0.5	-7.3	0.3	57° 25´1´´	16° 35´52´´
KLX03	-623	3	-72.2	0.5	-4.7	0.3	57° 25´1´´	16° 35´52´´
KLX03	-623	3	-73.0	0.5	-4.9	0.4	57° 25´1´´	16° 35´52´´
KLX04	-642	1	-11.4	0.5	-9.1	0.3	57° 25´38´´	16° 36´27´´
KLX04	-642	1	-63.1	0.5	-7.9	0.4	57° 25´38´´	16° 36´27´´
KLX04	-642	2	-59.7	0.5	-6.5	0.4	57° 25´38´´	16° 36´27´´
KLX04	-642	3	-11.7	0.5	n.a.		57° 25´38´´	16° 36´27´´
KLX04	-642	3	-65.1	0.4	-5.4	0.4	57° 25´38´´	16° 36´27´´
KLX04	-642	3	-65.8	0.6	-6.3	0.4	57° 25´38´´	16° 36´27´´
KLX04	-642	3	-75.7	0.5	-6.5	0.4	57° 25´38´´	16° 36´27´´
KLX09	-515	1	-25.8	0.5	-8.8	0.4	57° 25´44´´	16° 37´3´´
KLX09	-515	1	-9.7	0.5	-6.7	0.4	57° 25´44´´	16° 37´3´´
KLX07A	-231	1	-78.2	0.4	-6.9	0.4	57° 25´18´´	16° 37´29´´
KLX07A	-231	1	-72.0	0.4	-6.9	0.4	57° 25´18´´	16° 37´29´´
KLX07A	-231	1	-10.5	0.5	-5.8	0.4	57° 25´18´´	16° 37´29´´
KLX07A	-231	2	-53.9	0.5	-7.5	0.4	57° 25´18´´	16° 37´29´´
KLX07A	-231	2	-14.0	0.5	-7.5	0.3	57° 25´18´´	16° 37´29´´
KLX07A	-231	2	-13.6	0.5	-3.8	0.4	57° 25´18´´	16° 37´29´´
KLX13A	-367	1	-11.8	0.5	-5.8	0.3	57° 25´51´´	16° 35´1´´
KLX13A	-367	1	-31.4	0.7	-7.0	0.3	57° 25´51´´	16° 35´1´´
KLX13A	-367	2	-123.0	0.5	-6.4	0.3	57° 25´51´´	16° 35´1´´
KLX13A	-367	2	-28.4	0.5	-6.4	0.3	57° 25´51´´	16° 35´1´´
KLX13A	-367	2	-21.4	0.5	-7.0	0.3	57° 25´51´´	16° 35´1´´
KLX13A	-367	3	-120.1	0.5	-4.9	0.3	57° 25´51´´	16° 35´1´´
KLX13A	-367	3	-13.6	0.7	-12.7	0.3	57° 25´51´´	16° 35´1´´
KLX13A	-367	4	-115.2	0.5	-5.6	0.3	57° 25´51´´	16° 35´1´´
KLX13A	-367	4	-27.3	0.5	-7.5	0.3	57° 25´51´´	16° 35´1´´
KLX13A	-367	4	-14.4	0.5	-11.0	0.3	57° 25´51´´	16° 35´1´´
KLX13A	-367	5	-20.4	0.4	n.a.		57° 25´51´´	16° 35´1´´
KLX13A	-367	5	-26.0	0.5	-8.9	0.3	57° 25´51´´	16° 35´1´´
KLX13A	-367	5	-124.5	0.5	-4.9	0.3	57° 25´51´´	16° 35´1´´
KLX13A	-367	5	-29.5	0.5	-5.2	0.3	57° 25´51´´	16° 35´1´´
KLX13A	-367	5	-15.2	0.5	-6.9	0.3	57° 25´51´´	16° 35´1´´
KLX13A	-367	6	-9.7	0.7	-5.8	0.4	57° 25´51´´	16° 35´1´´
KLX13A	-367	6	-9.6	0.7	-3.8	0.4	57° 25´51´´	16° 35´1´´
KLX13A	-367	6	-91.8	0.6	-4.9	0.5	57° 25´51´´	16° 35´1´´
KLX13A	-367	6	-12.2	0.5	-5.4	0.4	57° 25´51´´	16° 35´1´´
KLX13A	-367	6	-17.3	0.5	-12.1	0.5	57° 25´51´´	16° 35´1´´
KLX13A	-367	7	-121.6	0.4	-3.9	0.5	57° 25´51´´	16° 35´1´´
KLX13A	-367	7	-122.1	0.4	-3.7	0.5	57° 25´51´´	16° 35´1´´
KLX13A	-367	7	-122.7	0.4	-4.2	0.4	57° 25´51´´	16° 35´1´´
KLX13A	-367	7	-122.4	0.4	-5.2	0.4	57° 25´51´´	16° 35´1´´
KLX13A	-367	7	-110.8	0.4	-5.1	0.4	57° 25´51´´	16° 35´1´´
KLX13A	-367	7	-29.7	0.4	-4.5	0.4	57° 25´51´´	16° 35´1´´
KLX13A	-367	7	-29.9	0.5	-10.9	0.4	57° 25´51´´	16° 35´1´´
KLX13A	-367	8	-113.8	0.4	-4.3	0.4	57° 25´51´´	16° 35´1´´
KLX13A	-367	8	-118.3	0.4	-4.4	0.4	57° 25´51´´	16° 35´1´´
KLX13A	-367	8	-109.0	0.4	-4.2	0.4	57° 25´51´´	16° 35´1´´
KLX13A	-367	8	-87.4	0.4	-4.6	0.4	57° 25´51´´	16° 35´1´´
KLX13A	-367	8	-23.8	0.5	-7.7	0.5	57° 25´51´´	16° 35´1´´
KLX13A	-367	8	-21.6	0.4	-9.8	0.4	57° 25´51´´	16° 35´1´´
KLX13A	-367	9	-100.1	0.4	-4.5	0.4	57° 25´51´´	16° 35´1´´

KLX13A	-367	9	-102.0	0.4	-4.3	0.4	57° 25´51´´	16° 35´1´´
KLX13A	-367	9	-104.1	0.4	-4.7	0.4	57° 25´51´´	16° 35´1´´
KLX13A	-367	9	-79.8	0.5	-5.5	0.5	57° 25´51´´	16° 35´1´´
KLX13A	-367	9	-28.0	0.4	-7.3	0.5	57° 25´51´´	16° 35´1´´
KLX13A	-367	9	-20.2	0.4	-6.4	0.4	57° 25´51´´	16° 35´1´´
KLX13A	-367	10	-91.1	0.4	-4.8	0.4	57° 25´51´´	16° 35´1´´
KLX13A	-367	10	-92.5	0.4	-5.6	0.4	57° 25´51´´	16° 35´1´´
KLX13A	-367	10	-91.2	0.4	-5.1	0.4	57° 25´51´´	16° 35´1´´
KLX13A	-367	10	-20.1	0.5	-8.1	0.5	57° 25´51´´	16° 35´1´´
KLX13A	-367	10	-11.0	0.5	-7.0	0.4	57° 25´51´´	16° 35´1´´
KLX13A	-367	10	-14.4	0.5	-11.9	0.4	57° 25´51´´	16° 35´1´´
KLX13A	-367	11	-111.2	0.4	-5.1	0.4	57° 25´51´´	16° 35´1´´
KLX13A	-367	11	-117.8	0.4	-5.6	0.4	57° 25´51´´	16° 35´1´´
KLX13A	-367	11	-119.0	0.4	-5.1	0.4	57° 25´51´´	16° 35´1´´
KLX13A	-367	11	-96.4	0.4	-6.1	0.4	57° 25´51´´	16° 35´1´´
KLX13A	-367	11	-21.2	0.5	-7.8	0.4	57° 25´51´´	16° 35´1´´
KLX13A	-367	11	-12.1	0.5	-6.1	0.5	57° 25´51´´	16° 35´1´´
KLX13A	-367	11	-14.9	0.5	-10.4	0.5	57° 25´51´´	16° 35´1´´
KLX13A	-370	1	-13.1	0.5	-8.4	0.3	57° 25´51´´	16° 35´1´´
KLX13A	-370	1	-12.3	0.5	-8.7	0.3	57° 25´51´´	16° 35´1´´
KLX13A	-370	1	-73.6	0.5	-4.7	0.3	57° 25´51´´	16° 35´1´´
KLX13A	-370	1	-73.5	0.5	-4.6	0.3	57° 25´51´´	16° 35´1´´
KLX13A	-370	2	-88.2	0.6	-5.3	0.3	57° 25´51´´	16° 35´1´´
KLX13A	-370	2	-104.3	0.5	-6.3	0.3	57° 25´51´´	16° 35´1´´
KLX13A	-370	2	-14.1	0.5	-18.1	0.4	57° 25´51´´	16° 35´1´´
KLX13A	-370	2	-77.7	0.5	-9.2	0.4	57° 25´51´´	16° 35´1´´
KLX13A	-370	3	-12.7	0.5	-4.6	0.3	57° 25´51´´	16° 35´1´´
KLX13A	-370	3	-11.9	0.5	-8.1	0.3	57° 25´51´´	16° 35´1´´
KLX13A	-370	3	-102.3	0.5	-5.0	0.3	57° 25´51´´	16° 35´1´´
KLX13A	-370	3	-103.9	0.5	-5.7	0.3	57° 25´51´´	16° 35´1´´
KLX13A	-370	3	-24.1	2.0	-11.9	0.3	57° 25´51´´	16° 35´1´´
KLX13A	-370	3	-89.0	0.5	-5.2	0.3	57° 25´51´´	16° 35´1´´
KLX13A	-370	4	-12.0	0.5	-6.9	0.3	57° 25´51´´	16° 35´1´´
KLX13A	-370	4	-103.4	0.5	-6.3	0.3	57° 25´51´´	16° 35´1´´
KLX13A	-370	4	-103.3	0.5	-6.2	0.3	57° 25´51´´	16° 35´1´´
KLX13A	-370	4	-102.3	0.5	-6.0	0.3	57° 25´51´´	16° 35´1´´
KLX18A	-447	1	-85.8	1.5	-6.1	0.3	57° 25´15´´	16° 36´8´´
KLX18A	-447	1	-41.1	0.5	-5.6	0.3	57° 25´15´´	16° 36´8´´
KLX18A	-447	1	-47.6	0.5	-4.8	0.3	57° 25´15´´	16° 36´8´´
KLX18A	-447	1	-98.3	0.5	-5.9	0.4	57° 25´15´´	16° 36´8´´
KLX18A	-447	2	-17.3	0.5	-7.4	0.4	57° 25´15´´	16° 36´8´´
KLX18A	-447	2	-16.3	0.5	-7.3	0.3	57° 25´15´´	16° 36´8´´
KLX18A	-447	2	-26.7	0.6	-4.7	0.3	57° 25´15´´	16° 36´8´´
KLX18A	-447	2	-27.3	0.5	-6.6	0.3	57° 25´15´´	16° 36´8´´
KLX18A	-447	2	-59.4	0.7	-7.7	0.3	57° 25´15´´	16° 36´8´´
KLX18A	-447	2	-45.8	0.5	-7.5	0.3	57° 25´15´´	16° 36´8´´
KLX18A	-447	3	-16.4	0.5	-7.8	0.4	57° 25´15´´	16° 36´8´´
KLX18A	-447	3	-64.9	1.0	-6.1	0.3	57° 25´15´´	16° 36´8´´
KLX18A	-447	4	-20.0	0.5	-9.6	0.3	57° 25´15´´	16° 36´8´´
KLX18A	-447	4	-68.5	0.5	-6.6	0.3	57° 25´15´´	16° 36´8´´
KLX18A	-447	4	-83.4	0.5	-7.5	0.3	57° 25´15´´	16° 36´8´´
KLX03	-378	1	-15.6	0.5	-6.7	0.3	57° 25´2´´	16° 35´55´´
KLX03	-378	1	-16.0	0.5	-7.6	0.3	57° 25´2´´	16° 35´55´´
KLX03	-378	1	-83.1	0.5	-7.9	0.4	57° 25´2´´	16° 35´55´´

KLX03	-378	2	-12.7	0.5	-7.2	0.3	57° 25´2´´	16° 35´55´´
KLX03	-378	2	-17.3	0.5	-7.5	0.3	57° 25´2´´	16° 35´55´´
KLX03	-378	2	-40.1	1.3	-8.0	0.6	57° 25´2´´	16° 35´55´´
KLX03	-378	3	-17.7	0.5	-7.9	0.3	57° 25´2´´	16° 35´55´´
KLX03	-378	3	-11.9	0.5	-7.1	0.3	57° 25´2´´	16° 35´55´´
KLX03	-378	3	-73.2	0.6	n.a.		57° 25´2´´	16° 35´55´´
KLX03	-378	3	-77.5	1.0	-5.1	0.3	57° 25´2´´	16° 35´55´´
KLX03	-378	3	-90.4	0.6	n.a.		57° 25´2´´	16° 35´55´´
KLX03	-378	3	-70.6	0.6	-6.4	0.3	57° 25´2´´	16° 35´55´´
KLX03	-378	4	-15.9	0.5	-6.7	0.3	57° 25´2´´	16° 35´55´´
KLX03	-378	4	-16.8	0.5	-7.3	0.3	57° 25´2´´	16° 35´55´´
KLX03	-378	4	-67.8	0.5	-7.4	0.3	57° 25´2´´	16° 35´55´´
KLX17A	-247	1	-15.6	0.4	-11.7	0.3	57° 25´34´´	16° 35´9´´
KLX17A	-247	1	-10.3	0.4	-6.1	0.3	57° 25´34´´	16° 35´9´´
KLX17A	-247	1	-15.3	0.5	-11.4	0.3	57° 25´34´´	16° 35´9´´
KLX17A	-247	2	-18.0	0.4	-6.5	0.2	57° 25´34´´	16° 35´9´´
KLX17A	-247	2	-10.2	0.4	-6.0	0.3	57° 25´34´´	16° 35´9´´
KLX17A	-247	2	-21.2	0.4	-3.7	0.3	57° 25´34´´	16° 35´9´´
KLX17A	-247	2	-7.4	0.5	-6.0	0.3	57° 25´34´´	16° 35´9´´
KLX17A	-247	3	-81.8	0.4	-7.3	0.3	57° 25´34´´	16° 35´9´´
KLX17A	-247	3	-11.2	0.4	-4.2	0.3	57° 25´34´´	16° 35´9´´
KLX17A	-247	3	-11.7	0.5	-4.7	0.3	57° 25´34´´	16° 35´9´´
KLX17A	-247	3	-16.5	0.4	-9.3	0.3	57° 25´34´´	16° 35´9´´
KLX03	-730	1	-6.9	0.4	-8.1	0.3	57° 24´60´´	16° 35´51´´
KLX03	-730	1	-64.6	0.5	-5.8	0.3	57° 24´60´´	16° 35´51´´
KLX03	-730	1	-45.5	0.4	-5.0	0.3	57° 24´60´´	16° 35´51´´
KLX03	-730	2	-6.4	0.4	-6.6	0.3	57° 24´60´´	16° 35´51´´
KLX03	-730	2	-27.5	0.4	-6.3	0.3	57° 24´60´´	16° 35´51´´
KLX03	-730	3	-5.9	0.4	-6.5	0.3	57° 24´60´´	16° 35´51´´
KLX03	-730	3	-68.5	0.5	-6.0	0.3	57° 24´60´´	16° 35´51´´
KLX03	-730	4	-6.8	0.5	-6.9	0.2	57° 24´60´´	16° 35´51´´
KLX03	-730	4	-6.4	0.5	-7.5	0.3	57° 24´60´´	16° 35´51´´
KLX08	-69	1	-34.7	0.4	-7.1	0.3	57° 25´34´´	16° 36´25´´
KLX08	-69	1	-11.2	0.4	-6.4	0.3	57° 25´34´´	16° 36´25´´
KLX08	-69	1	2.0	0.4	-6.6	0.3	57° 25´34´´	16° 36´25´´
KLX08	-69	1	-11.8	0.5	-8.6	0.2	57° 25´34´´	16° 36´25´´
KLX08	-69	2	-11.6	0.4	-7.5	0.2	57° 25´34´´	16° 36´25´´
KLX08	-69	2	-13.6	0.4	-7.8	0.3	57° 25´34´´	16° 36´25´´
KLX08	-69	3	2.6	0.4	-6.5	0.3	57° 25´34´´	16° 36´25´´
KLX08	-69	3	-13.7	0.4	-7.9	0.3	57° 25´34´´	16° 36´25´´
KLX08	-69	3	-13.7	0.5	-7.4	0.3	57° 25´34´´	16° 36´25´´
KLX08	-69	3	-11.1	0.4	-8.7	0.3	57° 25´34´´	16° 36´25´´
KLX08	-69	4	-11.7	0.4	-7.0	0.3	57° 25´34´´	16° 36´25´´
KSH01A	-200	1	-12.2	0.5	-9.0	0.5	57° 24´58´´	16° 40´41´´
KSH01A	-200	1	-4.0	0.5	-7.3	0.5	57° 24´58´´	16° 40´41´´
KSH01A	-200	1	1.7	0.5	-6.1	0.4	57° 24´58´´	16° 40´41´´
KSH01A	-200	1	-3.2	0.5	-3.9	0.4	57° 24´58´´	16° 40´41´´
KSH01A	-200	1	-12.4	0.5	-8.7	0.5	57° 24´58´´	16° 40´41´´
KSH01A	-200	2	-12.6	0.5	-5.7	0.4	57° 24´58´´	16° 40´41´´
KSH01A	-200	2	-5.2	0.5	-3.5	0.4	57° 24´58´´	16° 40´41´´
KSH01A	-200	2	1.1	0.5	-6.9	0.4	57° 24´58´´	16° 40´41´´
KSH01A	-200	2	-3.2	0.5	-4.7	0.4	57° 24´58´´	16° 40´41´´
KSH01A	-200	2	-7.1	0.5	-10.1	0.4	57° 24´58´´	16° 40´41´´
KSH01A	-200	3	-41.0	0.4	-4.7	0.4	57° 24´58´´	16° 40´41´´

KSH01A	-200	3	-22.4	0.4	-4.8	0.4	57° 24´58´´	16° 40´41´´
KSH01A	-200	3	0.5	0.5	-6.5	0.4	57° 24´58´´	16° 40´41´´
KSH01A	-200	3	-2.6	0.5	-3.8	0.4	57° 24´58´´	16° 40´41´´
KLX10	-89	1	2.2	0.4	-6.8	0.4	57° 25´11´´	16° 36´45´´
KLX10	-89	1	-2.1	0.5	-6.3	0.4	57° 25´11´´	16° 36´45´´
KLX10	-89	1	0.5	0.5	-5.8	0.4	57° 25´11´´	16° 36´45´´
KLX10	-89	2	1.6	0.4	-5.5	0.4	57° 25´11´´	16° 36´45´´
KLX10	-89	2	-2.7	0.4	-6.9	0.4	57° 25´11´´	16° 36´45´´
KLX10	-89	2	-0.3	0.5	-5.0	0.4	57° 25´11´´	16° 36´45´´
KLX10	-89	3	3.2	0.5	-7.4	0.4	57° 25´11´´	16° 36´45´´
KLX10	-89	3	2.8	0.4	-6.8	0.4	57° 25´11´´	16° 36´45´´
KLX10	-89	3	-3.3	0.5	-7.3	0.4	57° 25´11´´	16° 36´45´´
KLX10	-89	3	0.1	0.5	-5.0	0.4	57° 25´11´´	16° 36´45´´
KLX03	-170	1	-8.2	0.5	-5.6	0.3	57° 25´3´´	16° 35´56´´
KLX03	-170	1	-8.8	0.4	-8.4	0.3	57° 25´3´´	16° 35´56´´
KLX03	-170	1	-5.5	0.4	-7.6	0.3	57° 25´3´´	16° 35´56´´
KLX03	-170	2	-8.8	0.4	-8.4	0.3	57° 25´3´´	16° 35´56´´
KLX03	-170	3	4.8	0.4	-11.4	0.3	57° 25´3´´	16° 35´56´´
KLX03	-170	4	-7.2	0.5	-17.0	0.3	57° 25´3´´	16° 35´56´´
KLX03	-170	4	-16.4	0.4	-8.7	0.3	57° 25´3´´	16° 35´56´´
KLX03	-170	5	-8.1	0.5	-6.7	0.4	57° 25´3´´	16° 35´56´´
KLX03	-170	5	-8.0	0.5	-7.0	0.4	57° 25´3´´	16° 35´56´´
KLX03	-170	5	-7.1	0.5	-6.6	0.4	57° 25´3´´	16° 35´56´´
KLX03	-170	6	-9.8	0.4	-7.0	0.4	57° 25´3´´	16° 35´56´´
KLX03	-170	6	-9.0	0.5	-8.1	0.4	57° 25´3´´	16° 35´56´´
KLX03	-170	6	-10.3	0.4	-8.5	0.4	57° 25´3´´	16° 35´56´´
KLX03	-170	7	-9.8	0.5	-8.1	0.5	57° 25´3´´	16° 35´56´´
KLX03	-170	7	-9.5	0.5	-7.6	0.4	57° 25´3´´	16° 35´56´´
KLX03	-170	7	-11.7	0.5	-8.5	0.4	57° 25´3´´	16° 35´56´´
KLX09F	-47	1	1.0	0.5	-7.8	0.4	57° 25´43´´	16° 37´7´´
KLX09F	-47	1	0.4	0.5	-7.3	0.4	57° 25´43´´	16° 37´7´´
KLX09F	-47	1	-10.9	0.5	-6.2	0.4	57° 25´43´´	16° 37´7´´
KLX09F	-47	2	3.1	0.5	-7.2	0.4	57° 25´43´´	16° 37´7´´
KLX09F	-47	2	3.6	0.5	-7.4	0.4	57° 25´43´´	16° 37´7´´
KLX09F	-47	3	1.5	0.5	-7.4	0.4	57° 25´43´´	16° 37´7´´
KLX09F	-47	3	1.3	0.5	-6.3	0.4	57° 25´43´´	16° 37´7´´
KSH01B	-39	1	-10.4	0.5	-5.9	0.4	57° 24´60´´	16° 40´41´´
KSH01B	-39	1	-3.5	0.5	-10.3	0.4	57° 24´60´´	16° 40´41´´
KSH01B	-39	1	-3.2	0.5	-9.0	0.4	57° 24´60´´	16° 40´41´´
KSH01B	-39	2	1.2	0.5	-6.2	0.4	57° 24´60´´	16° 40´41´´
KSH01B	-39	2	-3.2	0.5	-7.9	0.4	57° 24´60´´	16° 40´41´´
KSH01B	-39	3	2.8	0.5	-4.6	0.4	57° 24´60´´	16° 40´41´´
KSH01B	-39	3	-3.1	0.5	-7.9	0.4	57° 24´60´´	16° 40´41´´
KLX15A	-190	1	-2.2	0.4	-7.9	0.3	57° 24´44´´	16° 36´10´´
KLX15A	-190	1	-15.1	0.4	-6.4	0.3	57° 25´43´´	16° 37´7´´
KLX15A	-190	1	-7.8	0.5	-5.2	0.3	57° 25´43´´	16° 37´7´´
KLX15A	-190	1	-1.0	0.4	-6.5	0.3	57° 25´43´´	16° 37´7´´
KLX15A	-190	2	-18.2	0.4	-7.3	0.3	57° 25´43´´	16° 37´7´´
KLX15A	-190	2	-8.3	0.5	-4.9	0.3	57° 25´43´´	16° 37´7´´
KLX15A	-190	2	0.0	0.4	-7.5	0.3	57° 25´43´´	16° 37´7´´
KLX15A	-190	2	-10.4	0.4	-8.2	0.3	57° 25´43´´	16° 37´7´´
KLX15A	-190	3	-1.5	0.5	-7.8	0.2	57° 25´43´´	16° 37´7´´
KLX15A	-190	3	-8.7	0.4	-8.0	0.3	57° 25´43´´	16° 37´7´´
KLX15A	-190	4	-8.8	0.5	-10.9	0.4	57° 25´43´´	16° 37´7´´

KLX15A	-190	5	-4.6	0.5	-8.7	0.4	57° 25´43´´	16° 37´7´´
KLX15A	-190	5	-1.2	0.5	-7.6	0.4	57° 25´43´´	16° 37´7´´
KLX09F	-27	1	-18.4	0.7	-6.4	0.4	57° 25´43´´	16° 37´6´´
KLX09F	-27	1	-18.5	0.7	-6.9	0.4	57° 25´43´´	16° 37´6´´
KLX09F	-27	1	-18.0	0.7	-5.8	0.4	57° 25´43´´	16° 37´6´´
KLX09F	-27	2	-17.8	0.7	-6.0	0.4	57° 25´43´´	16° 37´6´´
KLX09F	-27	2	-17.5	0.7	-6.5	0.4	57° 25´43´´	16° 37´6´´
KLX20A	-182	1	-6.3	0.5	-3.6	0.4	57° 25´13´´	16° 34´40´´
KLX20A	-182	1	-5.7	0.5	-5.9	0.4	57° 25´13´´	16° 34´40´´
KLX20A	-182	1	-7.7	0.5	-6.0	0.4	57° 25´13´´	16° 34´40´´
KLX20A	-182	2	-6.2	0.5	-5.2	0.4	57° 25´13´´	16° 34´40´´
KLX20A	-182	2	-7.9	0.5	-7.2	0.4	57° 25´13´´	16° 34´40´´
KLX20A	-182	2	-10.9	0.5	-7.0	0.4	57° 25´13´´	16° 34´40´´
KLX20A	-182	3	-7.8	0.5	-6.2	0.4	57° 25´13´´	16° 34´40´´
KLX20A	-182	3	-5.2	0.5	-6.1	0.4	57° 25´13´´	16° 34´40´´
KLX20A	-182	3	-8.1	0.5	-6.1	0.4	57° 25´13´´	16° 34´40´´
KLX20A	-182	4	-7.1	0.5	-3.8	0.4	57° 25´13´´	16° 34´40´´
KLX20A	-182	4	-5.9	0.4	-6.9	0.4	57° 25´13´´	16° 34´40´´
KLX20A	-182	4	-14.5	0.4	-6.5	0.4	57° 25´13´´	16° 34´40´´
KLX20A	-182	4	-7.5	0.4	-6.1	0.4	57° 25´13´´	16° 34´40´´
KLX10	-180	1	-7.3	0.5	-4.6	0.4	57° 25´11´´	16° 36´45´´
KLX10	-180	1	-13.8	0.5	-6.2	0.4	57° 25´11´´	16° 36´45´´
KLX10	-180	1	-8.2	0.5	-8.8	0.4	57° 25´11´´	16° 36´45´´
KLX10	-180	1	-11.4	0.4	-7.8	0.4	57° 25´11´´	16° 36´45´´
KLX10	-180	2	-7.4	0.5	-5.3	0.4	57° 25´11´´	16° 36´45´´
KLX10	-180	2	-7.2	0.5	-4.4	0.4	57° 25´11´´	16° 36´45´´
KLX10	-180	2	-6.4	0.5	-4.2	0.4	57° 25´11´´	16° 36´45´´
KLX10	-180	3	-7.6	0.5	-12.0	0.4	57° 25´11´´	16° 36´45´´
KLX10	-180	3	-9.4	0.5	-5.7	0.4	57° 25´11´´	16° 36´45´´
KLX10	-180	3	-5.8	0.5	-6.8	0.4	57° 25´11´´	16° 36´45´´
KLX09D	6	1	-19.3	0.5	-7.7	0.4	57° 25´44´´	16° 37´7´´
KLX09D	6	1	-19.2	0.5	-6.8	0.4	57° 25´44´´	16° 37´7´´
KLX09D	6	1	-9.4	0.4	-7.1	0.4	57° 25´44´´	16° 37´7´´
KLX09D	6	2	-18.1	0.5	-6.8	0.5	57° 25´44´´	16° 37´7´´
KLX09D	6	2	-13.7	0.5	-6.8	0.5	57° 25´44´´	16° 37´7´´
KLX09D	6	2	-11.0	0.4	-8.1	0.4	57° 25´44´´	16° 37´7´´
KLX04	-80.7	1	-9.4	0.5	-7.3	0.4	57° 25´36´´	16° 36´26´´
KLX04	-80.7	1	-11.0	0.5	-8.4	0.4	57° 25´36´´	16° 36´26´´
KLX04	-80.7	1	-8.5	0.5	-7.1	0.4	57° 25´36´´	16° 36´26´´
KLX04	-80.7	1	-5.4	0.5	-7.1	0.4	57° 25´36´´	16° 36´26´´
KLX04	-80.7	2	-10.1	0.5	-8.0	0.4	57° 25´36´´	16° 36´26´´
KLX04	-80.7	2	-8.7	0.5	-6.8	0.4	57° 25´36´´	16° 36´26´´
KLX04	-80.7	2	-6.9	0.5	-6.8	0.4	57° 25´36´´	16° 36´26´´
KLX04	-80.7	2	-8.4	0.5	-8.2	0.4	57° 25´36´´	16° 36´26´´
KLX09D	3	1	-9.2	0.5	-8.6	0.4	57° 25´44´´	16° 37´7´´
KLX09D	3	1	-9.1	0.5	-8.1	0.4	57° 25´44´´	16° 37´7´´
KLX09D	3	2	-9.9	0.4	-7.4	0.5	57° 25´44´´	16° 37´7´´
KLX09D	3	2	-9.0	0.4	-8.6	0.4	57° 25´44´´	16° 37´7´´
KLX09D	3	3	-9.5	0.5	-8.1	0.4	57° 25´44´´	16° 37´7´´
KLX09D	3	4	-10.6	0.5	-8.5	0.4	57° 25´44´´	16° 37´7´´
KLX09D	3	4	-9.3	0.5	-8.9	0.5	57° 25´44´´	16° 37´7´´
KLX09D	3	4	-9.5	0.4	-7.5	0.5	57° 25´44´´	16° 37´7´´
KLX21B	-116	1	-19.5	0.5	-6.6	0.5	57° 25´5´´	16° 37´55´´
KLX21B	-116	1	-18.6	0.5	-8.4	0.4	57° 25´5´´	16° 37´55´´

KLX21B	-116	1	-13.5	0.5	-7.3	0.4	57° 25´5´´	16° 37´55´´
KLX21B	-116	2	-18.0	0.4	-7.9	0.5	57° 25´5´´	16° 37´55´´
KLX21B	-116	2	-4.2	0.5	-8.7	0.5	57° 25´5´´	16° 37´55´´
KLX21B	-116	2	-3.0	0.5	-8.0	0.4	57° 25´5´´	16° 37´55´´
KLX21B	-116	2	-5.1	0.5	-9.0	0.4	57° 25´5´´	16° 37´55´´
KLX21B	-116	2	-8.5	0.5	-9.7	0.4	57° 25´5´´	16° 37´55´´
KLX21B	-116	3	-5.3	0.5	-9.7	0.4	57° 25´5´´	16° 37´55´´
KLX21B	-116	3	-5.8	0.5	-9.6	0.5	57° 25´5´´	16° 37´55´´
KLX21B	-116	3	-5.1	0.5	-8.7	0.4	57° 25´5´´	16° 37´55´´
KLX21B	-116	3	-5.8	0.5	-8.8	0.4	57° 25´5´´	16° 37´55´´
KSH01A	-916	1	-6.1	0.5	-7.7	0.5	57° 24´52´´	16° 40´38´´
KSH01A	-916	1	-6.6	0.5	-7.9	0.4	57° 24´52´´	16° 40´38´´
KSH01A	-916	1	-5.6	0.5	-8.2	0.4	57° 24´52´´	16° 40´38´´
KLX03	-859	1	-5.4	0.5	-7.0	0.4	57° 24´59´´	16° 35´50´´
KLX03	-859	1	-6.8	0.5	-6.9	0.5	57° 24´59´´	16° 35´50´´
KLX03	-859	2	-5.7	0.5	-7.8	0.4	57° 24´59´´	16° 35´50´´
KLX03	-859	2	-6.6	0.5	-8.0	0.5	57° 24´59´´	16° 35´50´´
KLX03	-859	3	-6.4	0.5	-8.6	0.5	57° 24´59´´	16° 35´50´´
KLX04	-944	1	-7.1	0.5	-9.1	0.4	57° 25´38´´	16° 36´29´´
KLX04	-944	1	-7.1	0.5	-9.0	0.4	57° 25´38´´	16° 36´29´´
KLX04	-944	2	-7.0	0.5	-9.2	0.4	57° 25´38´´	16° 36´29´´
KLX04	-944	2	-7.8	0.5	-7.2	0.4	57° 25´38´´	16° 36´29´´
KLX04	-944	3	-8.1	0.5	-9.0	0.4	57° 25´38´´	16° 36´29´´
KLX04	-944	3	-6.4	0.5	-9.0	0.4	57° 25´38´´	16° 36´29´´
KLX09F	11	1	-23.3	0.5	-5.4	0.4	57° 25´43´´	16° 37´5´´
KLX09F	11	2	-15.2	0.5	-6.5	0.4	57° 25´43´´	16° 37´5´´
KLX09F	11	2	-10.1	0.5	-5.2	0.4	57° 25´43´´	16° 37´5´´
KLX09C	-2	1	-1.3	0.5	-8.3	0.4	57° 25´44´´	16° 37´6´´
KLX09C	-2	1	-4.2	0.5	-8.1	0.4	57° 25´44´´	16° 37´6´´
KLX04	-897	1	-9.1	0.5	-9.4	0.4	57° 25´38´´	16° 36´29´´
KLX04	-897	1	-9.5	0.5	-7.5	0.4	57° 25´38´´	16° 36´29´´
KLX04	-897	2	-9.7	0.5	-9.1	0.4	57° 25´38´´	16° 36´29´´
KLX04	-897	3	-9.0	0.5	-9.7	0.4	57° 25´38´´	16° 36´29´´
KLX04	-897	3	-11.0	0.5	-9.3	0.4	57° 25´38´´	16° 36´29´´
KLX03	-249	1	-9.6	0.5	-8.2	0.4	57° 25´3´´	16° 35´56´´
KLX03	-249	1	-8.4	0.5	-8.2	0.4	57° 25´3´´	16° 35´56´´
KLX03	-249	1	-8.3	0.5	-9.5	0.4	57° 25´3´´	16° 35´56´´
KLX03	-249	1	-8.8	0.5	-9.5	0.4	57° 25´3´´	16° 35´56´´
KLX03	-249	1	-9.1	0.5	-9.6	0.4	57° 25´3´´	16° 35´56´´
KLX03	-249	2	-9.5	0.5	-8.1	0.4	57° 25´3´´	16° 35´56´´
KLX03	-249	2	-6.5	0.5	-7.6	0.4	57° 25´3´´	16° 35´56´´
KLX03	-249	2	-6.7	0.5	-8.1	0.4	57° 25´3´´	16° 35´56´´
KLX10	-208	1	-3.0	0.5	-8.3	0.3	57° 25´11´´	16° 36´45´´
KLX10	-208	1	-6.3	0.4	-8.3	0.3	57° 25´11´´	16° 36´45´´
KLX10	-208	1	-7.6	0.5	-7.6	0.3	57° 25´11´´	16° 36´45´´
KLX10	-208	1	-4.8	0.4	-8.7	0.3	57° 25´11´´	16° 36´45´´
KLX10	-208	2	-3.3	0.4	-6.9	0.3	57° 25´11´´	16° 36´45´´
KLX10	-208	2	-9.2	0.5	-7.6	0.3	57° 25´11´´	16° 36´45´´
KLX10	-208	2	-5.9	0.4	-8.3	0.3	57° 25´11´´	16° 36´45´´
KLX04	-871	1	-7.8	0.4	-8.5	0.3	57° 25´38´´	16° 36´29´´
KLX04	-871	1	-7.9	0.4	-8.3	0.3	57° 25´38´´	16° 36´29´´
KLX04	-871	2	-8.1	0.5	-7.0	0.3	57° 25´38´´	16° 36´29´´
KLX15A	-740	1	-4.3	0.5	-5.3	0.3	57° 24´28´´	16° 36´3´´
KLX15A	-740	2	-3.9	0.4	-9.1	0.3	57° 24´28´´	16° 36´3´´

KLX15A	-740	1	-5.6	0.4	-6.9	0.3	57° 24´28´´	16° 36´3´´
KLX15A	-740	2	-4.7	0.5	-8.0	0.3	57° 24´28´´	16° 36´3´´
KLX15A	-740	1	-4.6	0.4	-10.0	0.3	57° 24´28´´	16° 36´3´´
KLX15A	-740	2	-4.3	0.4	-7.7	0.3	57° 24´28´´	16° 36´3´´
KLX11D	-56	1	-5.5	0.7	-7.0	0.4	57° 25´13´´	16° 34´50´´
KLX11D	-56	1	-2.3	0.7	-12.4	0.5	57° 25´13´´	16° 34´50´´
KLX11D	-56	1	-4.4	0.7	-11.2	0.4	57° 25´13´´	16° 34´50´´
KLX11D	-56	1	-5.1	0.7	-7.5	0.4	57° 25´13´´	16° 34´50´´
KLX11D	-56	1	-6.3	0.7	-7.2	0.5	57° 25´13´´	16° 34´50´´
KLX11D	-56	2	-4.7	0.7	-7.9	0.4	57° 25´13´´	16° 34´50´´
KLX11D	-56	2	-4.1	0.7	-12.8	0.4	57° 25´13´´	16° 34´50´´
KLX11D	-56	2	-4.0	0.7	-11.2	0.4	57° 25´13´´	16° 34´50´´
KLX11D	-56	2	-6.8	0.7	-6.9	0.5	57° 25´13´´	16° 34´50´´
KLX11D	-56	2	-7.4	0.7	-9.1	0.5	57° 25´13´´	16° 34´50´´
KLX11D	-56	3	-3.7	0.7	-13.4	0.4	57° 25´13´´	16° 34´50´´
KLX11D	-56	3	-5.3	0.7	-10.9	0.4	57° 25´13´´	16° 34´50´´
KLX11D	-56	3	-8.7	0.7	-7.6	0.4	57° 25´13´´	16° 34´50´´
KLX21B	-131	1	-3.4	0.4	-7.3	0.4	57° 25´5´´	16° 37´55´´
KLX21B	-131	1	-4.1	0.4	-8.4	0.4	57° 25´5´´	16° 37´55´´
KLX21B	-131	1	-4.7	0.4	-8.9	0.4	57° 25´5´´	16° 37´55´´
KLX21B	-131	1	-7.9	0.5	-8.2	0.4	57° 25´5´´	16° 37´55´´
KLX21B	-131	1	-5.5	0.5	-7.7	0.4	57° 25´5´´	16° 37´55´´
KLX21B	-131	1	-14.3	0.4	-8.1	0.4	57° 25´5´´	16° 37´55´´
KLX21B	-131	2	-3.9	0.5	-8.7	0.4	57° 25´5´´	16° 37´55´´
KLX21B	-131	2	-3.8	0.5	-8.3	0.4	57° 25´5´´	16° 37´55´´
KLX21B	-131	2	-3.6	0.5	-8.6	0.4	57° 25´5´´	16° 37´55´´
KLX21B	-131	2	-6.7	0.5	-8.2	0.4	57° 25´5´´	16° 37´55´´
KLX21B	-131	2	-8.1	0.5	-8.4	0.4	57° 25´5´´	16° 37´55´´
KLX21B	-131	2	-13.3	0.4	-7.8	0.4	57° 25´5´´	16° 37´55´´
KLX21B	-131	3	-21.8	0.4	-7.3	0.4	57° 25´5´´	16° 37´55´´
KLX21B	-131	3	-15.0	0.5	-8.3	0.4	57° 25´5´´	16° 37´55´´
KLX21B	-131	3	-4.4	0.5	-7.5	0.4	57° 25´5´´	16° 37´55´´
KLX21B	-131	3	-4.7	0.5	-7.9	0.4	57° 25´5´´	16° 37´55´´
KLX04	-647	1	-15.0	0.6	-8.2	0.2	57° 25´38´´	16° 36´27´´
KLX04	-647	1	-100.4	0.5	-8.8	0.2	57° 25´38´´	16° 36´27´´
KLX04	-647	1	-92.6	0.5	-8.9	0.2	57° 25´38´´	16° 36´27´´
KLX04	-647	2	-16.1	0.6	-8.4	0.2	57° 25´38´´	16° 36´27´´
KLX04	-647	2	-100.5	0.5	-7.9	0.2	57° 25´38´´	16° 36´27´´
KLX04	-647	2	-92.3	0.6	-8.2	0.2	57° 25´38´´	16° 36´27´´
KLX04	-647	2	-99.7	0.5	-9.2	0.2	57° 25´38´´	16° 36´27´´
KSH01A	-206	1	-18.8	0.4	-7.9	0.2	57° 24´58´´	16° 40´41´´
KSH01A	-206	1	-16.1	0.4	-8.2	0.2	57° 24´58´´	16° 40´41´´
KSH01A	-206	2	-18.7	0.5	-6.1	0.2	57° 24´58´´	16° 40´41´´
KSH01A	-206	2	-45.5	0.4	-5.3	0.2	57° 24´58´´	16° 40´41´´
KSH01A	-206	3	-18.2	0.4	-7.2	0.3	57° 24´58´´	16° 40´41´´
KSH01A	-206	3	-18.0	0.5	-7.9	0.2	57° 24´58´´	16° 40´41´´
KSH01A	-206	3	-16.9	0.4	-6.7	0.2	57° 24´58´´	16° 40´41´´
KLX11E	-79	1	-12.5	0.5	-5.2	0.2	57° 25´13´´	16° 34´51´´
KLX11E	-79	1	-7.7	0.5	-6.4	0.2	57° 25´13´´	16° 34´51´´
KLX11E	-79	1	-6.9	0.5	-10.5	0.2	57° 25´13´´	16° 34´51´´
KLX11E	-79	2	-11.9	0.7	-9.4	0.2	57° 25´13´´	16° 34´51´´
KLX11E	-79	2	-7.9	0.7	-6.3	0.2	57° 25´13´´	16° 34´51´´
KLX11E	-79	2	-15.3	0.7	-7.0	0.2	57° 25´13´´	16° 34´51´´
KSH01A	-205	1	-18.5	0.7	-5.5	0.2	57° 24´58´´	16° 40´41´´

KSH01A	-205	2	-16.1	0.7	-6.4	0.2	57° 24´58´´	16° 40´41´´
KLX09C	-76	1	-9.1	0.7	-5.5	0.2	57° 25´43´´	16° 37´7´´
KLX09C	-76	1	-16.7	0.7	-7.4	0.2	57° 25´43´´	16° 37´7´´
KLX09C	-76	1	-10.0	0.7	-9.7	0.2	57° 25´43´´	16° 37´7´´
KLX09C	-76	2	-12.2	0.7	-4.2	0.2	57° 25´43´´	16° 37´7´´
KLX09C	-76	2	-14.9	0.7	-8.0	0.3	57° 25´43´´	16° 37´7´´
KLX09C	-76	2	-14.5	0.5	-9.0	0.2	57° 25´43´´	16° 37´7´´
KLX08	-328	1	-18.9	0.5	-5.0	0.2	57° 25´30´´	16° 36´20´´
KLX08	-328	1	-19.9	0.5	-8.6	0.2	57° 25´30´´	16° 36´20´´
KLX08	-328	1	-25.6	0.5	-9.0	0.2	57° 25´30´´	16° 36´20´´
KLX08	-328	2	-22.5	0.5	-5.0	0.2	57° 25´30´´	16° 36´20´´
KLX08	-328	2	-19.4	0.5	-9.0	0.2	57° 25´30´´	16° 36´20´´
KLX08	-328	2	-12.3	0.5	-7.0	0.2	57° 25´30´´	16° 36´20´´

n.a.=not analysed

Supplementary Table 2. Groundwater data.
(n.a.=not analysed)

Borehole	Depth (mid-section) (m.a.s.l.)	$\delta^{18}O$ (‰SMOW)	$\delta^{13}C_{DIC}$ (‰V-PDB)	HCO_3^- (mg L^{-1})	SO_4^{2-} (mg L^{-1})	DOC (mg L^{-1})
KLX01	-163.26	-10.6	-15.6	277	105	9.0
KLX01	-257.06	-11.5	n.a.	83.0	48.0	1.5
KLX01	-672.95	-13.3	n.a.	24.0	351	1.2
KLX03	-170.82	-11.5	-17.0	328	36.6	21.0
KLX03	-379.85	-13.0	-27.1	189	127	13.0
KLX03	-922.45	-12.2	n.a.	7.70	758	1.4
KLX04	-854.86	-14.1	n.a.	10.1	475	2.2
KLX04	-491.94	-15.2	-20.2	33.1	102	2.1
KLX04	-486.52	-15.1	n.a.	51.4	104	2.2
KLX04	-944.38	-13.8	n.a.	8.48	845	0.5
KLX05	-204.84	-12.1	-14.9	194	146	7.0
KLX05	-204.84	-11.8	-15.0	185	155	6.0
KLX05	-549.56	-12.2	n.a.	11.6	415	n.a.
KLX06	-221.18	-11.3	-16.5	224	40.6	3.5
KLX06	-475.27	-13.3	-18.3	71.7	423	3.3
KLX06	-475.27	-13.0	-18.8	86.3	376	3.6
KLX06	-475.27	n.a.	n.a.	8.14	11.5	0.1
KLX07A	-569.69	-11.6	n.a.	24.2	317	2.0
KLX08	-390.71	-15.6	n.a.	32.0	133	2.5
KLX08	-504.54	-15.2	n.a.	20.5	145	1.8
KLX08	-500.90	-16.0	n.a.	20.2	135	1.7
KLX08	-500.90	-15.8	n.a.	20.6	131	2.0
KLX08	-500.90	-16.0	n.a.	20.0	135	2.1
KLX08	-539.39	-14.7	n.a.	18.0	146	1.6
KLX08	-539.39	-14.6	n.a.	16.6	149	1.6
KLX10	-338.43	-10.4	-15.0	71.1	176	2.5
KLX10	-338.43	-10.5	-15.9	69.0	175	2.6
KLX10	-676.19	-11.0	n.a.	26.6	193	2.1
KLX10	-676.19	-11.2	-22.7	25.4	208	1.8
KLX11A	-542.07	-14.2	-19.8	42.6	130	2.3
KLX12A	-501.12	-12.3	n.a.	15.3	354	2.0
KLX12A	-501.12	-12.3	n.a.	14.2	344	2.3
KLX12A	-501.12	-12.2	n.a.	13.9	348	2.1
KLX13A	-408.01	-15.9	-16.6	73.9	36.8	2.7
KLX13A	-474.99	-15.1	n.a.	83.7	47.3	2.6
KLX15A	-192.74	-12.7	-18.3	50.0	49.0	1.7
KLX15A	-192.74	-12.2	-17.5	70.4	86.6	2.0
KLX15A	-192.74	-11.9	n.a.	84.5	90.4	2.5
KLX15A	-467.22	-10.8	n.a.	14.1	425	1.5
KLX15A	-469.27	-10.7	n.a.	21.3	375	3.8
KLX15A	-469.27	-10.9	-18.1	17.8	377	1.6
KLX17A	-342.32	-14.4	-16.8	122.0	22.9	3.4
KLX18A	-452.87	-14.2	-19.2	50.2	139	3.1
KLX18A	-452.87	-14.1	-20.4	41.2	144	3.1
KLX19A	-413.86	-13.9	-17.8	36.3	122	1.5
KLX19A	-413.86	-14.0	-17.3	30.2	223	1.2
KLX19A	-413.86	-14.0	n.a.	28.0	110	1.4
KLX19A	-413.86	-14.0	n.a.	25.9	116	1.4
KLX19A	-624.78	-12.7	-15.9	55.3	196	3.1
KLX20A	-183.32	n.a.	n.a.	107	58.4	3.5
KLX20A	-183.32	-13.8	-16.9	107	59.1	3.5
KLX27A	-562.96	-15.0	n.a.	13.1	108	1.4
HLX01	-56.19	-10.9	n.a.	233	63.8	5.3
HLX03	-46.69	-10.9	n.a.	204	21.5	4.0

HLX06	-46.42	-10.6	n.a.	249	23.5	7.4
HLX10	-29.25	-10.8	-16.1	219	49.2	n.a.
HLX14	-42.17	-11.2	-17.2	223	53.6	n.a.
HLX20	-54.46	-11.5	-16.8	198	36.9	3.1
HLX22	-57.00	-11.3	-15.2	221	53.1	n.a.
HLX23	-51.94	-11.3	-16.4	224	59.7	n.a.
HLX24	-59.62	-10.8	-12.5	121	29.6	n.a.
HLX28	-52.67	-10.9	-16.2	265	35.8	n.a.
HLX30	-60.04	-13.5	-13.4	146	71.5	n.a.
HLX34	-50.17	-11.2	-16.5	297	65.4	n.a.
HLX35	-89.75	-11.4	-16.1	244	59.1	n.a.
HLX37	-69.00	-11.3	-15.4	226	28.0	n.a.
HLX38	-28.73	-11.9	-14.8	194	53.9	n.a.
HLX39	-137.87	-11.2	-17.4	226	10.1	n.a.

Supplementary Table 3. SIMS analyses of pyrite (* from [1]). For coordinates, see supplementary table 1.

Borehole	Depth (m.a.s.l.)	Crystal	$\delta^{34}S$ (‰V-CDT)	± ‰
KLX04	-647	1	6.65	0.12
KLX04	-647	1	8.10	0.12
KLX04	-647	1	9.20	0.12
KLX04	-647	2	8.19	0.12
KLX04	-647	2	9.60	0.12
KLX04	-647	2	8.68	0.12
KLX04	-647	3	13.1	0.12
KLX04	-647	3	14.0	0.12
KLX04	-647	3	17.4	0.12
KLX04	-647	4	7.30	0.12
KLX04	-647	4	6.72	0.12
KLX04	-647	4	2.69	0.14
KLX04	-642	1	-3.79	0.12
KLX04	-642	1	-2.93	0.12
KLX04	-642	1	-0.08	0.12
KLX04	-642	2	16.5	0.12
KLX04	-642	2	16.9	0.13
KLX13A	-367	1	20.6	0.16
KLX13A	-367	1	24.0	0.12
KLX13A	-367	1	24.4	0.12
KLX13A	-367	1	24.6	0.12
KLX13A	-367	2	25.7	0.13
KLX13A	-367	2	25.5	0.12
KLX13A	-367	2	23.4	0.12
KLX13A	-367	2	25.5	0.12
KLX13A	-367	3	26.1	0.12
KLX13A	-367	3	27.1	0.12
KLX13A	-367	3	27.8	0.12
KLX13A	-367	4	25.8	0.12
KLX13A	-367	4	25.9	0.12
KLX13A	-367	4	27.5	0.12
KLX13A	-367	5	26.2	0.12
KLX13A	-367	5	27.0	0.12
KLX13A	-367	5	27.2	0.12
KLX13A	-367	5	26.7	0.12
KLX13A	-367	6	17.6	0.19
KLX13A	-367	6	24.4	0.12
KLX13A	-367	7	26.7	0.12
KLX13A	-367	7	27.2	0.12
KLX13A	-369	1	40.4	0.12
KLX13A	-369	1	40.4	0.12
KLX13A	-369	1	29.0	0.12
KLX13A	-369	2	0.80	0.12
KLX13A	-369	2	4.88	0.12
KLX13A	-369	2	15.1	0.12
KLX13A	-369	3	26.2	0.12
KLX13A	-369	3	26.7	0.12
KLX13A	-369	3	27.3	0.12
KLX13A	-370	1	23.6	0.12
KLX13A	-370	1	27.1	0.12

KLX13A	-370	1	25.6	0.12
KLX13A	-370	2	20.9	0.12
KLX13A	-370	2	22.9	0.12
KLX13A	-370	2	26.5	0.12
KLX13A	-370	3	25.9	0.12
KLX13A	-370	3	25.6	0.12
KLX13A	-370	3	25.5	0.12
KLX13A	-370	4	22.0	0.12
KLX13A	-370	4	26.8	0.12
KLX13A	-370	4	25.3	0.12
KLX07A	-230	1	7.61	0.12
KLX07A	-230	1	10.8	0.12
KLX07A	-230	1	0.40	0.13
KLX07A	-230	2	11.4	0.12
KLX07A	-230	2	-19.5	0.12
KLX07A	-230	2	-22.1	0.13
KLX17A	-346	1	24.8	0.12
KLX17A	-346	1	24.7	0.12
KLX17A	-346	1	18.5	0.12
KLX17A	-346	2	26.1	0.12
KLX17A	-346	3	3.74	0.12
KLX17A	-346	3	2.49	0.12
KLX17A	-346	3	2.47	0.12
KLX17A	-346	3	2.72	0.12
KLX17A	-346	4	25.6	0.12
KLX17A	-346	4	26.2	0.12
KLX17A	-346	5	1.81	0.12
KLX17A	-346	5	2.15	0.12
KLX17A	-346	5	2.06	0.12
KLX03	-623	1	11.8	0.13
KLX03	-623	1	14.5	0.12
KLX03	-623	1	13.9	0.12
KLX03	-623	1	13.0	0.12
KLX03	-623	2	-6.39	0.30
KLX03	-623	2	27.3	0.54
KLX03	-623	2	32.4	0.13
KLX03	-623	3	16.5	0.12
KLX03	-623	3	46.8	0.16
KLX03	-623	3	34.7	0.12
KLX03	-623	4	12.3	0.12
KLX03	-623	4	12.7	0.12
KLX03	-623	4	26.9	0.24
KLX04	-528	1	16.7	0.12
KLX04	-528	1	18.6	0.12
KLX04	-528	1	20.0	0.12
KLX04	-528	2*	9.60	0.10
KLX04	-528	2*	12.0	0.10
KLX04	-528	2*	9.00	0.10
KLX04	-528	2*	9.30	0.10
KLX04	-528	3*	-7.20	0.10
KLX04	-528	3*	-11.1	0.10
KLX04	-528	3*	3.80	0.10
KLX18A	-447	1	22.7	0.12
KLX18A	-447	1	23.1	0.12

KLX18A	-447	1	23.6	0.12
KLX18A	-447	2	2.08	0.12
KLX18A	-447	2	-8.76	0.12
KLX18A	-447	2	23.0	0.12
KLX18A	-447	3*	16.5	0.40
KLX18A	-447	3*	24.8	0.40
KLX18A	-447	3*	26.0	0.40
KLX18A	-447	4*	15.1	0.40
KLX18A	-447	4*	24.9	0.40
KLX18A	-447	4*	20.8	0.40

Supplementary Table 4. Compounds and corresponding characteristic fragments detected with ToF-SIMS within the AOM-calcites. The compound and fragments assignments was conducted through comparison with references spectra [2-5]. A representative ToF-SIMS spectrum is shown in Fig. 4 of the main article.

Compound (abbreviation; formula; exact mass)	Measured $m\,z^{-1}$ (+)	Calculated $m\,z^{-1}$ (+)	Tentative formula	Measured $m\,z^{-1}$ (-)	Calculated $m\,z^{-1}$ (-)	Tentative formula
17α(H),21β(H)-Norhopane (norhopane; $C_{29}H_{50}$; 398.39)	149.14	149.13	$[C_{11}H_{17}]^+$			
	163.15	163.15	$[C_{12}H_{19}]^+$			
	177.17	177.16	$[C_{13}H_{21}]^+$			
	191.18	191.18	$[C_{14}H_{23}]^+$			
	205.20	205.20	$[C_{15}H_{25}]^+$			
	219.22	219.21	$[C_{16}H_{27}]^+$			
	259.25	259.24	$[C_{19}H_{31}]^+$			
	367.35	367.34	$[C_{27}H_{43}]^+$			
	369.36	369.35	$[C_{27}H_{45}]^+$			
	383.39	383.37	$[C_{28}H_{47}]^+$			
	397.39	397.38	$[M-H]^+$			
Hop-17(21)-ene or Hop-22(29)-ene (diploptene) ($C_{30}H_{50}$; 410.39)	149.14	149.13	$[C_{11}H_{17}]^+$			
	161.14	161.13	$[C_{12}H_{17}]^+$			
	177.17	177.16	$[C_{13}H_{21}]^+$			
	189.17	189.16	$[C_{14}H_{21}]^+$			
	191.18	191.18	$[C_{14}H_{23}]^+$			
	203.19	203.18	$[C_{15}H_{23}]^+$			
	205.20	205.20	$[C_{15}H_{25}]^+$			
	217.20	217.20	$[C_{16}H_{25}]^+$			
	219.22	219.21	$[C_{16}H_{27}]^+$			
	231.21	231.21	$[C_{17}H_{27}]^+$			
	367.35	367.34	$[C_{27}H_{43}]^+$			
	369.36	369.35	$[C_{27}H_{45}]^+$			
	393.36	393.35	$[C_{29}H_{45}]^+$			
	395.37	395.37	$[C_{29}H_{47}]^+$			
	409.39	409.38	$[M-H]^+$			
	411.40	411.40	$[M+H]^+$			
Dialkyl glycerol diether (DAGE; $C_{37}H_{72}O_3$; 564.55)	267.28	267.27	$[C_{18}H_{35}O]^+$			
	311.28	311.29	$[C_{20}H_{39}O_2]^+$			
	327.29	327.29	$[C_{20}H_{39}O_3]^+$			
	547.53	547.55	$[M-H_2O]^+$			
	565.54	565.56	$[M+H]^+$			
Dialkyl glycerol diether (DAGE, $C_{35}H_{68}O_3$; 536.52)	239.25	239.24	$[C_{16}H_{31}O]^+$			
	283.26	283.26	$[C_{18}H_{35}O_2]^+$			
	311.28	311.29	$[C_{20}H_{39}O_2]^+$			
	327.29	327.29	$[C_{20}H_{39}O_3]^+$			
	519.49	519.51	$[M-H_2O]^+$			
	537.51	537.52	$[M+H]^+$			
Dialkyl glycerol diether (DAGE, $C_{33}H_{68}O_3$; 512.52)	227.23	227.24	$[C_{16}H_{31}O]^+$			
	285.27	285.28	$[C_{18}H_{37}O_2]^+$			
	301.26	301.27	$[C_{18}H_{37}O_3]^+$			
	495.51	495.51	$[M-H_2O]^+$			
	513.53	513.52	$[M+H]^+$			
Dialkyl glycerol diether (DAGE, $C_{29}H_{58}O_3$; 454.44)	255.23	255.23	$[C_{16}H_{31}O_2]^+$			
	257.24	257.25	$[C_{16}H_{33}O_2]^+$			
	271.24	271.23	$[C_{16}H_{31}O_3]^+$			
	437.44	437.44	$[M-H_2O]^+$			
	455.43	455.45	$[M+H]^+$			
Diacylglycerolester (DG 16:0/18:0; $C_{37}H_{72}O_5$; 596.54)	239.25	239.24	$[C_{16}H_{31}O]^+$	255.23	255.23	$[C_{16}H_{31}O_2]^-$
	267.26	267.27	$[C_{18}H_{35}O]^+$	283.26	283.26	$[C_{18}H_{35}O_2]^-$
	313.28	313.27	$[C_{19}H_{37}O_3]^+$			
	341.32	341.31	$[C_{21}H_{41}O_3]^+$			
	579.54	579.54	$[M-H_2O]^+$			
Diacylglycerolester (DG; 15:0/17:0 $C_{35}H_{68}O_5$; 568.51)	225.23	225.22	$[C_{15}H_{29}O]^+$	241.22	241.22	$[C_{15}H_{29}O_2]^-$
	253.26	253.25	$[C_{17}H_{33}O]^+$	269.25	269.25	$[C_{17}H_{33}O_2]^-$
	299.26	299.26	$[C_{19}H_{37}O_3]^+$			
	327.29	327.29	$[C_{20}H_{39}O_3]^+$			
	551.50	551.50	$[M-H_2O]^+$			

Supplementary Table 5. Chemistry of chlorite (in mixed-layer clay) from EDS analyses. All data in wt.%. Scanning electron microscope analyses revealed compositional zonation of the crystals (variation in back-scatter intensities of different parts of the crystals) as well as presence of fine-grained minerals at grain-boundaries within the crystals (Supplementary Fig. 2). Energy-dispersive spectroscopy analyses (EDS) revealed that these minerals are mainly clay minerals, dominantly chlorite (as revealed by Raman spectroscopy). The presence of Na, K and Ca indicates that chlorite is not pure but mixed-layered (e.g. corrensite: chlorite interstratified with smectite or vermiculite). Pyrite were found together with the clay minerals and in a few cases hematite.

Sample	KLX15A: -199m	KLX15A: -199m	KLX13A: -367m	KSH01B: -39m	KSH01B: -39m
Na_2O	0.15	0.14	0.16	0.68	0.55
MgO	16.4	16.2	12.1	17.4	15.5
Al_2O_3	13.1	12.2	12.0	12.5	12.2
SiO_2	30.3	29.4	33.4	32.7	34.3
K_2O	0.20	0.21	b.d.	0.30	2.06
CaO	2.40	2.13	2.32	1.95	2.50
MnO	0.88	0.75	0.46	0.52	0.36
FeO	20.0	17.7	19.1	14.9	12.1
Total	83.4	78.6	79.5	81.0	79.5

b.d.=below detection (0.1 wt.%). In “total”, crystal-bound water is excluded.

Supplementary Table 6. Fluid inclusion data of calcite with inclusions large enough for certain microthermometric determination.

**Borehole**	**Depth (m.a.s.l.)**	**Crystal**	**Type of fluid inclusion**	**T_h* °C**	**T_{fm} °C**	**T_m** °C**	**Salinity wt. % NaCl eq.**
KLX04	-642	3	aqueous liquid	<50	-26 to -30	-1.4	2.4
	-642	3	aqueous liquid	<50	-26 to -30	-1.4	2.4
KLX10	-180	1	aqueous liquid	<50	-24	-4.1	6.6
	-180	1	aqueous liquid	<50	-24	-0.5	0.9
	-180	3	aqueous liquid	<50	-35	-9.9	13.8
	-180	3	aqueous liquid	<50	-35	-3.8	6.2
	-180	3	aqueous liquid	<50	-35	-3.1	5.1
	-180	3	aqueous liquid	<50	-35	-3.5	5.7

Supplementary Notes

Supplementary Note 1: Raman spectroscopy

Analyses were carried out on calcite within the same epoxy-mounts used for SIMS-analyses. After microscopic investigation of all crystals, samples KLX03:-170 m, KLX15A: -190 m, KSH01A: -200 m, KLX13A: -367 m, KLX10:-89 m, KLX09F:-47 m and KLX13A: -369 were selected for analysis with Raman spectroscopy. The calcites showed no specific organic peaks in the crystal matrix, neither in crystals with AOM-related $\delta^{13}C$-signature, nor in crystals with methanogenesis-related $\delta^{13}C$-signature. Secondary minerals at the grain-boundaries were dominated by chlorite (supplementary Fig. 2 shows clinochlore in the spectrum), probably in mixed-layer clay as shown by the detectable amounts of Na, Ca and K in the EDS analyses (see supplementary table 5), and some hematite. Similar mineralogy was found in the fine-grained coating at the calcite-wall rock contact (base of crystal in supplementary Fig. 2).

Supplementary Note 2: Fluid inclusions

Fluid inclusions were searched for in all samples analysed with SIMS (except KLX07A:-231 m). The fluid inclusions were all single-phase all-liquid aqueous inclusions, either randomly spread out within the crystals. All-liquid aqueous inclusions were also present as planar groups of secondary inclusions that outline healed microfractures within the crystals. The size of the primary inclusions was mostly less than 5 µm, and accurate microthermometry measurement could therefore only be carried out in a few samples (Supplementary Table 6). Other inclusions were too small to allow certain optical determinations of ice melting and several crystals did not contain any inclusions at all. Slow undisturbed crystal growth at low degree of oversaturation in the aqueous solution results in such fluid inclusion-poor crystals[7]. The inclusions found display a diversity of shapes, most typically round or irregular, but also with a poorly developed rectangular shape (Supplementary Fig. 3). No leakage of the fluid inclusions was observed. For the single-phased inclusions, homogenisation temperature could not be measured, but entrapment at temperatures below 50˚C is suggested for such inclusions[8]. The salinities of the inclusions are calculated from the final ice melting temperature (T_m) of the inclusions using data for the NaCl-H_2O system[9]. To avoid metastable ice melting this temperature was measured after inducing an artificial vapour bubble by heating and stretching the inclusions. Melting of ice occurred between -0.5˚ and -9.9˚C (n=8) which corresponds to a salinity of 0.9 to 13.8 mass % NaCl eq in the two samples measured, one AOM-type calcite (KLX04:-642 m) and one calcite with neither methanogenesis- nor AOM-related $\delta^{13}C$ values and with varied $\delta^{18}O$ values (KLX10:-180 m). The AOM-calcite showed marine-brackish type salinities (2.4 wt.% NaCl eq.) whereas the other sample showed a salinity ranging from 0.9 to 13.8 wt.% NaCl eq. with most values in the span 5.1-6.6 wt.% NaCl eq. The different salinities of the latter sample either reflect the varying waters related to the different growth zones within the calcite, and/or mixing of different water such as brine with brackish to fresh water based on the spread in salinities. The calcites sampled in this study clearly post-date calcite rich in two-phased fluid inclusions with brine type salinities and homogenisation temperature above 50°C[10,11], dated to the Paleozoic era[12] (see example of the relative temporal relation between these two calcite types in Supplementary Fig. 4) .

Supplementary Note 3: Physical modelling

Methane is typically formed via biological activity in fractures in the uppermost layers of the crystalline bedrock. The biological activity is concentrated within about 200 m below the surface. Further down methane production is low. Methane is oxidised and deposited as calcite on fracture walls at depths of about 250-750 m. It is unclear how the methane can be transported down to these depths as, typically, surface waters have a lower density than waters at the greater depths. Advection, i.e. active flow of surface water downwards in fractures may occur under different circumstances but it is unclear whether advection is able to account for downward methane transport in general. The other candidate to explain the transport of the methane to form the observed calcite deposits is diffusion.

A differential equation that may be used to describe the transport of methane in the case explained above is the so called advection-diffusion equation. As boundary conditions we assume that biological activity produces a steady concentration of methane in the range 10^{-2} -10^{0} L m^{-3} at a ’source’ depth of 100 m. We also assume that at a depth of 500 m all methane is converted in to calcite and methane concentration is thus zero. We further assume that methane transport occurs along water filled two-dimensional fracture planes that have a width in the order of 1 mm. The diffusion constant, D, for methane in water is $D \approx 1.5 \times 10^{-9}$ m^2 s^{-1} [13]. Because D is roughly constant and water is incompressible we can write the advection-diffusion equation as

$$\frac{\partial \eta}{\partial t} = D\nabla^2\eta + \vec{v} \cdot \nabla\eta, \qquad (1)$$

where η is the methane concentration that depend on time, t, and the downward space component x, that runs from $x = 0$ at the methane source at 100 m depth to the methane sink at $x = 400$ at 500 m depth, ∇ is the vector differential operator (the gradient) and $\vec{v}$ is the downward flow velocity of the water in the fracture.

In a steady state η does not depend on time t, which means that the left-hand side of Eq. (1) vanish. If we first consider the case when the water in a fracture is stagnant, i.e. $\vec{v} = 0$. Then we need to find a solution to $D\nabla^2\eta = 0$ that fulfil the boundary conditions $\eta(0) = c$ (c is here the methane concentration at the source) and $\eta(400) = 0$. The solution is, $\eta(x) = c(L - x)/L$, where $L = 400$ is the distance from source to sink. The methane flux, J, at steady-state is given by $J = -Dd\eta/dx = Dc/L$.

If we now insert a source methane concentration of $c \approx 10^{-1}$ L m^{-3} (l=litre), we obtain $J \propto 10^{-13}$ L $m^{-2}s^{-1}$. If we furthermore consider a 1 m portion of a fracture of width 10^{-3} m we obtain a flux of the order of 10^{-8} L $year^{-1}$ of methane. It thus takes roughly 100 million years for 1 litre of methane per meter of fracture length to be transported down to the sink depth. This result can be compared to opposite scenario when diffusion can be neglected and only advection is contributing significantly to methane transport. In this case the flux is $J = cv$. If again $c \approx 10^{-1}$ Lm^{-3} a flow velocity large than 10^{-12} m s^{-1} would be enough for advection to dominate over diffusion.

In conclusion, it seems to be little doubt that advection is completely dominating the methane transport.

Supplementary References

1 Drake, H., Åström, M., Tullborg, E.-L., Whitehouse, M. J. & Fallick, A. E. Variability of sulphur isotope ratios in pyrite and dissolved sulphate in granitoid fractures down to 1 km depth - evidence for widespread activity of sulphur reducing bacteria. *Geochim. Cosmochim. Acta* **102**, 143–161 (2013).

2 Leefmann, T. *et al.* Spectral characterization of ten cyclic lipids using time-of-flight secondary ion mass spectrometry. *Rapid Commun. Mass Sp.* **27**, 565-581 (2013).

3 Leefmann, T. *et al.* Biomarker imaging of single diatom cells in a microbial mat using time-of-flight secondary ion mass spectrometry (ToF-SIMS). *Org. Geochem.* **57**, 23-33 (2013).

4 Heim, C., Sjövall, P., Lausmaa, J., Leefmann, T. & Thiel, V. Spectral characterization of eight glycerolipids and their detection in natural samples using time-of-flight secondary ion mass spectrometry. *Rapid Commun. Mass Sp.* **23**, 2741-2753 (2009).

5 Siljeström, S., Hode, *et al.* Detection of organic biomarkers in crude oils using ToF-SIMS. *Org. Geochem.* **40**, 135-143 (2009).

6 Downs, R. T. The RRUFF Project: an integrated study of the chemistry, crystallography, Raman and infrared spectroscopy of minerals. *Program and Abstracts of the 19th General Meeting of the International Mineralogical Association in Kobe, Japan.* **O03-13** (2006).

7 Roedder, E. *Fluid Inclusions, Reviews in Mineralogy*. Vol. 12 (Mineralogical Society of America, Washington D.C. USA, 1984).

8 Goldstein, R. H. Fluid inclusions in sedimentary and diagenetic systems. *Lithos* **55**, 159-193 (2001).

9 Bodnar, R. J. Introduction to aqueous-electrolyte fluid inclusions. In *Fluid Inclusions: Analysis and Interpretation.* Vol. 32 (eds I. Samson, A. Anderson, & D. Marshall) 81-99 (Mineralogical Association of Canada, 2003).

10 Drake, H. & Tullborg, E.-L. Paleohydrogeological events recorded by stable isotopes, fluid inclusions and trace elements in fracture minerals in crystalline rock, Simpevarp area, SE Sweden. *Appl. Geochem.* **24**, 715-732 (2009).

11 Milodowski, A. E. *et al.* Application of mineralogical, petrological and geochemical tools for evaluating the palaeohydrogeological evolution of the PADAMOT study sites. *PADAMOT Project Technical Report WP2*, 206 (UK Nirex Ltd, Harwell, UK, 2005).

12 Drake, H., Tullborg, E.-L. & Page, L. Distinguished multiple events of fracture mineralisation related to far-field orogenic effects in Paleoproterozoic crystalline rocks, Simpevarp area, SE Sweden. *Lithos* **110**, 37-49 (2009).

13 http://www.engineeringtoolbox.com/diffusion-coefficients-d_1404.html.
